# Mongolian medicine theory-based multidimensional evaluation of toxicity mitigation in Hezi-processed Caowu jointly mediated by powder dosage form and small dose

**DOI:** 10.3389/fphar.2025.1679105

**Published:** 2025-10-01

**Authors:** Jing Wang, Liyuan Bao, Ying Zhao, Liangliang Song, Wenting Zu, Jiasheng Wang, Hongshuang Chi, Yichen Li, Hong Du

**Affiliations:** School of Chinese Materia Medica, Beijing University of Chinese Medicine, Beijing, China

**Keywords:** *Aconiti kusnezoffii* Radix, Chebulae Fructus, dosage form, toxicity and effect, alkaloid, p38, JNK MAPK

## Abstract

**Backgroud:**

Caowu (CW, *Aconitum kusnezoffii* Reichb.) is a well-known Mongolian medicine with the effects of dispelling cold and relieving pain. In China, it is widely used in the prevention and treatment of rheumatoid arthritis (RA). However, its cardiotoxicity and neurotoxicity seriously restrict its clinical application. Different from the use of CW as a decoction after being boiled in water in TCM, in Mongolian medicine, CW is processed using Hezi (HZ, dry ripe fruit of *Terminalia chebula* Retz. and *T. chebula* Retz. var. *tomentella* Kurt.) decoction or combined with HZ to prepare pills or powders, and administered at a small dosage, thereby ensuring medication safety. This way of medication is a useful experience of the minority. Thus, multi-dimensional research (quantitation of alkaloid + *in vitro* + *in vivo* + mechanism) is critical to elucidate the characteristics of the Mongolian ethnic group’s detoxification experience. We investigated the content of alkaloids, the anti-inflammatory and analgesic effects, the cardiorenal-hepatic toxicities, and the oxidative stress levels in both powder and decoction of raw CW (SCW) and HZ-processed CW (HCW), with a focus on the toxicity mechanisms in H9c2 cells (specifically for the powder dosage).

**Methods:**

HPLC was used to quantify the content of main metabolites in SCW and HCW (in powder or decoction form). The pharmacological effects were evaluated using *in vivo* animal models (xylene-induced inflammation and formalin-induced pain). The toxicity of SCW and HCW was assessed *via* electrocardiographic analysis, histological analysis, and biochemical analysis. Subsequently, *in vitro* toxicity mechanism studies were conducted on H9c2 cells using techniques such as MTT, fluorescent probes, ELISA, and Western blotting. Additionally, the involvement of the p38/JNK signaling pathway in their cardiotoxicity was verified by treating cells with p38/JNK pathway inhibitors.

**Results:**

Using HPLC, we found that both the use of HZ as an excipient for processing CW and decocting CW with water affect the content of alkaloids in CW. In the xylene-induced ear edema model, SCW powder, SCW decoction, and HCW powder showed significant anti-inflammatory effects by regulating inflammation-related factors (IL-1β, IL-6, and IL-10); in contrast, HCW decoction did not show significant anti-inflammatory effects compared with the model group. In the formalin-induced pain model, both SCW and HCW exerted analgesic effects to varying degrees by regulating pain-related factors (5-HT and PGE2). In the toxicity study, SCW powder exhibited the strongest toxicity to the heart; in contrast, SCW decoction had the weakest toxicity, while HCW powder and HCW decoction fell in between. Furthermore, the types of arrhythmia induced by SCW powder were most complex. In addition, the cardiotoxicity of SCW was closely related to oxidative stress. Cell experiments showed that SCW induced H9c2 cell damage, which HCW partially mitigated by regulating the p38/JNK pathway.

**Conclusion:**

In conclusion, compared with SCW, HCW reduces toxicity while exerting pharmacological effects in powder dosage form. This attenuation is linked to a reduction in oxidative stress, inhibition of p38/JNK phosphorylation, and regulation of mitochondrial apoptosis-related protein expression. This finding advances our understanding of the coexistence of toxicity and efficacy in clinical application of CW.

## Graphical Abstract

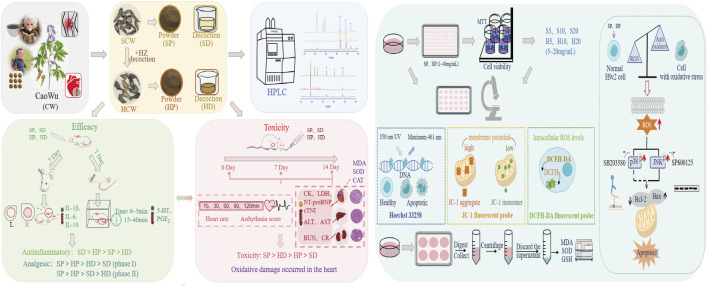

## 1 Introduction

Caowu (CW), the dried tuberous root of *Aconitum kusnezoffii* Reichb., is an important Mongolian medicine with the effects of dispelling cold and relieving pain and has been used clinically in China for thousands of years. CW is traditionally used for the treatment of rheumatoid arthritis (RA) and joint pain. Many classic Mongolian medicine formulas containing CW (such as Garidi Wuwei Wan, Naru Sanwei Wan and Zhachong Shisanwei Wan) were frequently used by the public to relieve pain and inflammation (Wu et al., 2021).

However, due to the narrow treatment window of CW, its clinical use is often accompanied with some toxicity reactions. Among them, cardiotoxicity is the main clinical manifestation of CW poisoning. After poisoning, patients often present with symptoms such as abnormal electrocardiogram (ECG), palpitation and arrhythmia, and the heart failure induced by malignant arrhythmias is the primary cause of death after CW poisoning (Sheth et al., 2015; Ye et al., 2019). Today, there is still no specific antidote. CW has a complex composition of plant metabolites, notably diterpenoid alkaloids, which are recognized as both the primary bioactive and toxic metabolites (Liu et al., 2017b; Wu and Bao, 2024). The mechanism of cardiac toxicity caused by CW is complex, involving oxidative stress injury, apoptosis, calcium overload, ion channels (such as sodium ion, potassium ion, and calcium ion channels), energy metabolism, and the expression of apoptosis-related proteins (Zhou et al., 2013; Sun et al., 2014; Zhou et al., 2021; Gao et al., 2022). Among them, oxidative stress is one of the important mechanisms leading to cardiotoxicity, and cardiotoxicity will further aggravate oxidative stress and form a vicious circle. When oxidative stress is out of balance, excessive reactive oxygen species (ROS) are produced in the body; these ROS damage the structure of myocardial cells, affect the function of myocardial cells, activate the apoptotic pathway, and lead to cardiotoxicity. When the heart is damaged by toxicity, the metabolic process in cardiomyocytes changes, resulting in more ROS and further aggravating oxidative stress (Ma et al., 2018; Xia et al., 2021).

In fact, CW is rarely used directly. Reducing the toxicity of CW by processing is an indispensable step in its safe clinical application. Diester alkaloids have poor stability and are prone to hydrolysis reactions. In Traditional Chinese Medicine (TCM), steaming or boiling are commonly used to process CW, and this practice is widely recognized to promote the hydrolysis of diester alkaloids. Thus, heating-induced hydrolysis is established as one of the core detoxification principles for CW in TCM (Liu et al., 2025). Hezi (HZ), the dried fruit of *Terminalia chebula* Retz., has a therapeutic effect on various ailments (such as moderating the potent toxicity of toxic drugs) and is the “King of All Medicines.” Among Mongolian medicine clinical prescriptions that contain CW, more than 50% also include HZ (Wang P. et al., 2025). What’s more, when Mongolian medicine uses CW, pills and powders are the mainstream formulations (due to better toxicity control and dosage accuracy). According to traditional Mongolian medical theory, CW is typically processed using HZ decoction to yield HCW or directly combined with HZ (Li et al., 2019; Li H. et al., 2021), and they are often used in the form of pills or powders, such as Naru Sanwei Wan (Guo et al., 2023). The reason is that the clinical medication of Mongolian medicine focuses on convenient storage and small doses. It has the advantages of the “Three Smalls” (small toxicity, small side effects, and small dosage), the “Three Effects” (high efficacy, quick effect, and long-lasting effect), and the “Five Conveniences” (convenient collection, preparation, carrying, administration, and storage) (Wang and Du, 2018). This is determined by its nomadic living environment and the course characteristics of RA “chronic, long-term and flexible treatment.”

When CW is processed with HZ decoction, its acute toxicity decreases. Our previous study found that the median lethal dose (LD_50_) of HCW in mice was 1.1690 g/kg, while that of SCW was 0.7009 g/kg (Liu et al., 2015a). The medication experience of processing CW with HZ decoction, guided by Mongolian medical theory, has been validated by long-term practice. However, its modern scientific detoxification principle remains to be fully elucidated. The existing research speculates that the tannins in HZ react with the alkaloids in CW to form a water-insoluble complex (Yang et al., 2013). These complexes exhibit more stable release and absorption in the body, avoiding the toxicity caused by high blood concentration and playing a delayed-release effect (Liu et al., 2015b). On the other hand, HZ exhibits strong pharmacological activity, especially in the cardiovascular system (Wang et al., 2024). While previous research has focused on the differences between SCW and processed CW (SCW processed with HZ decoction or compatible with HZ) using chemical and pharmacological methods (Yuan et al., 2022; Guo, 2024), the specific differences in chemical composition, efficacy, and toxicity between their powder and decoction dosage forms remain unexplored. Beyond the complexation theory, the detoxification mechanism also involves multifaceted pharmacological interventions. In cellular studies, tannins and triterpenes in HZ can reduce the toxicity caused by active metabolites in SCW. This attenuated effect is related to the expression of the TRPV1 channel (transient receptor potential vanilloid type 1 channel, a non-selective ion channel that mediates calcium ion influx and participates in energy metabolism, cell proliferation, and apoptosis), cardiac metabolic enzymes, and cytochrome enzymes (Li et al., 2023; Song et al., 2024). Researchers have investigated the toxicity and mechanism of SCW at the genetic level and found that it could significantly affect the MAPK pathway in mice (Zhang et al., 2008). Another study used the network pharmacology method to explore the effect of HZ on reducing the neurotoxicity of aconitine. The results showed that it may play a role by regulating the oxidative stress response of the nervous system and the MAPK signaling pathway (Tao et al., 2023b). Notably, SCW can lead to increased intracellular ROS release and mitochondrial damage (Han et al., 2020), and ROS generation leads to oxidative stress, which in turn activates key targets in the MAPK pathway to lead to cell apoptosis. In addition, we found that mesaconitine has a high affinity for multiple proteins in the MAPK signaling pathway through molecular docking. Similarly, multiple triterpenoids in HZ also have the potential to bind to these proteins (Supplementary Appendix SI). Thus, it is worth further exploring whether the attenuation effect of HCW is related to the MAPK signaling pathway.

In the present study, first we used high-performance liquid chromatography (HPLC) to determine the contents of six ester-type alkaloids in different dosage forms (powder, water decoction) of SCW (powder: SP; water decoction: SD) and HCW (powder: HP; water decoction: HD). Then we compared their efficacy and toxicity in laboratory animals. Additionally, by using H9c2 cardiomyocytes, we compared the cardiomyocyte toxicity and oxidative damage levels of SP and HP. Finally, after screening the appropriate concentration for inducing toxic effects, we used Western blot experiments to verify whether the related effects were related to MAPK signaling pathway and to explore the potential toxic mechanism of SCW and HCW and the possible attenuation mechanism of HCW. This fills the gap in research on the differences in efficacy and toxicity of different processed products of CW in different dosage forms, reveals the potential molecular mechanism of cardiotoxicity, and provides a theoretical basis for the safe medication of CW.

## 2 Materials and methods

### 2.1 Chemicals and reagents

Acetonitrile, methyl alcohol and tetrahydrofuran of HPLC grade were supplied by Fisher Chemicals (Pittsburg, United States). Ammonium acetate and ammonia water were purchased from Tianjin Fuchen Chemical Reagent Co., Ltd., glacial acetic acid, ethyl acetate, isopropanol, and xylene from Tianjin Zhiyuan Chemical Reagent Co., Ltd., absolute ethanol from Tianjin Damao Chemical Reagent Factory, and sodium carboxymethyl cellulose (CMC-Na) from Tianjin Bailunsi Biotechnology Co., Ltd. (all in Tianjin, China). The water used for liquid chromatography was provided by Wahaha Co., Ltd. (Hangzhou, China). All other reagents were of analytical grade. The 4% paraformaldehyde was purchased from Wuhan Xavier Biotechnology Co., Ltd. (Wuhan, China).

The reference standards (HPLC greater than 98%) of benzoylaconine, benzoylhypacoitine, benzoylmesaconine, aconitine, hypaconitine, and mesaconitine were purchased from Chengdu Desite Biotechnology Co., Ltd. (Chengdu, China).

Phosphate-buffered saline (PBS) was purchased from Beijing Solarbio Science & Technology Co., Ltd. (Beijing, China). Dulbecco’s Modified Eagle Medium (DMEM, high glucose), fetal bovine serum (FBS), penicillin-streptomycin mixture, and 0.05% trypsin were all obtained from Dalian Meilun Biotechnology Co., Ltd. (Dalian, China). 3-(4,5-Dimethylthiazol-2-yl)-2,5-diphenyltetrazolium bromide (MTT) was procured from Beijing Lanbo Lide Biotechnology Co., Ltd. (Beijing, China). Dimethyl sulfoxide (DMSO) was sourced from Sigma-Aldrich (Steinheim, Germany).

Rat H9c2 cell line was obtained from Cell Resource Center, Institute of Basic Medical Sciences, Chinese Academy of Medical Sciences (Beijing, China).

### 2.2 Plant materials

Aconiti Kusnezoffii Radix [Ranunculaceae; *Aconitum kusnezoffii* Reichb.] was purchased from Beijing Huamiao Traditional Chinese Medicine Engineering Technology Development Center (Beijing, China). Chebulae Fructus [Combretaceae; *T. chebula* Retz.] was purchased from Beijing Shuangqiao Yanjing Chinese Herbal Pieces Factory (Beijing, China), and they were authenticated by Prof. Jingjuan Wang from the Beijing University of TCM. The voucher specimens were deposited in the School of Chinese Materia Medica, Beijing University of TCM, China.

### 2.3 Preparation of SCW and HZ-decoction processed SCW (HCW)

After removing impurities, CW was washed with water, then dried to obtain SCW. According to the 2020 edition of the “Processing Specification of Inner Mongolia Mongolian Medicine Decoction Pieces” (Inner Mongolia Autonomous Region Drug Administration, 2020), the following is a description of the processing technology of HCW: Take the SCW, put it into the HZ decoction, soak at room temperature for 3–5 days, flip 3 to 5 times a day. When taken out, it makes the tongue feel slightly numb, then dry at low temperature. Specifically, HZ (60.0 g) was soaked in deionized water (600 mL, at a weight-to-volume ratio of 1:10) for a duration of 1 h, followed by a boiling process lasting 1 h. Subsequently, Hezi decoction was added to SCW (200 g) in a beaker. Hezi decoction was replaced every day, and SCW was stirred during soaking. Soaking for 5 days is a process that our team studied to meet the limited requirements of ester alkaloids in relevant standards.

### 2.4 HPLC analysis of SCW and HCW

#### 2.4.1 Preparation of sample solution

For SCW and HCW powder: 2.00 g of the powder of sample (40 mesh size) was accurately weighed and placed in a conical flask with a stopper. 3 mL of ammonia test solution was added, and then 50 mL of a 1:1 (v:v) mixture of isopropanol and ethyl acetate was precisely added, weighed and ultrasonically treated with a power of 300 W, a frequency of 40 kHz, and the water temperature below 25 °C for 30 min, cooled. Following the extraction process, isopropanol-ethyl acetate (1:1, v:v) was added to compensate for any weight loss experienced during extraction, after which the mixture was filtered. Next, 25 mL of the resulting filtrate was accurately measured and evaporated to dryness under decreasedpressure at 40 °C. The residue was dissolved in 5 mL of 0.01% (w:v) hydrochloric acid solution (in ethanol) and filtered through a 0.22 µm membrane filter.

For SCW and HCW water decoction: 2.50 g of the sample was accurately weighed and placed in a conical bottle. Water was added at a ratio of 1:10 (w:v, sample:water), and soaked for 30 min, then heated under reflux for 1 h, filtered, and the filtrate was collected. Water was again added to the filter residue at a ratio of 1:10 (w:v, sample:water) to continue decocting for 30 min, followed by filtration. The two filtrates were combined, then concentrated to 10 mL by rotary evaporation at below 40 °C to prepare a 250 mg/mL water decoction sample. The sample was filtered through a 0.22 µm membrane filter and injected into an HPLC system for analysis.

Identification of the metabolites obtained from the extraction has been reported in previous studies by our research group. The results of UPLC-Orbitrap-MS showed that the representative metabolites in SCW and HCW were characterized in HESI ± mode (Zhi et al., 2020a). According to the Chinese Pharmacopoeia, benzoylmesaconine, benzoylaconine, benzoylhypaconine, mesaconitine, hypaconitine, and aconitine are key indicators of toxicity control/efficacy evaluation. Therefore, we optimized the method and quantified these alkaloids.

#### 2.4.2 Preparation of standard solution

A mixed standard stock solution containing benzoylmesaconine, benzoylaconine, mesaconitine, benzoylhypaconine, hypaconitine, and aconitine was first prepared. This stock solution was then diluted with 0.01% hydrochloric acid-ethanol to obtain eight mixed reference solutions with suitable concentrations, which were used for developing calibration curves.

The calibration curves were constructed by plotting the peak areas of the reference standards against their corresponding concentrations. For detection, the injection volume of the mixed reference solutions was set at 10 μL.

#### 2.4.3 Chromatography conditions

HPLC analyses were conducted utilizing a Waters 2695 HPLC system (Waters Corporation, Milford, MA, United States) equipped with a Waters 2998 Photodiode Array Detector (PDA), and Empower 3 chromatography workstation software (Waters Corporation). The separation procedure was performed using an Agilent ZORBAX SB-C_18_ column (4.6 × 250 mm, 5 μm). Chromatographic conditions for SCW and HCW powder: Phase A was acetonitrile-tetrahydrofuran (25:15, v/v), and phase B was 0.1 mol/L ammonium acetate in water. The gradients of mobile phases were as follows: 0.00 min, 15% A and 85% B; 35.00 min, 21.6% A and 78.4% B; 50.00 min, 27% A and 73% B; 60.00 min, 35% A and 65% B; 65.00 min, 15% A and 85% B. The analysis was operated at a flow rate of 1 mL/min and a column temperature of 25 °C, with an injection volume of 10 μL. Detection was performed at 235 nm.

Chromatographic conditions for SCW and HCW water decoction: Phase A was acetonitrile and phase B was 40 mmol/L ammonium acetate buffer (adjusted to pH 10 with ammonia test solution). The gradients of mobile phases were as follows: 0.00 min, 15% A and 85% B; 45.00 min, 60% A and 40% B; 60.00 min, 60% A and 40% B; 65.00 min, 15% A and 85% B. The chromatographic column model and other operating conditions were the same as those used for the determination of powder.

#### 2.4.4 HPLC method validation

The HPLC conditions were validated according to the guidelines set forth by the Chinese Pharmacopoeia 2020. The parameters of linearity, accuracy, precision, and stability were assessed through the analysis of samples and various concentrations of mixed reference solutions. Variability was quantified by conducting analyses on six replicates. The relative standard deviation (RSD) was computed to assess precision, stability and repeatability. Additionally, six samples of SCW were prepared and analyzed on the same day to assess repeatability. Stability was examined at ambient temperature, with analyses conducted at intervals of 0, 4, 8, 12, 18, and 24 h. The recovery was determined by calculating the recovery percentages of benzoylmesaconine, benzoylaconine, mesaconitine, benzoylhypaconine, hypaconitine, and aconitine in the spiked samples. Six parallel samples were prepared for each concentration.

### 2.5 Animals and treatments

Sixty-three Male Sprague-Dawley rats (42 days, 200 ± 10 g), and 160 male Institute of Cancer Research (ICR) mice (4 weeks, 18–24 g) were obtained from Beijing Si Pei Fu, China (No. SCXK (Beijing) 2019-0010). All animal experiments were conducted with the approval of the animal ethics committee of Beijing University of Chinese Medicine (No. BUCM-4-2023022102-1018, No.BUCM-4-2023022103-1019). Prior to commencing the experiment, all animals were allowed to adapt to the environment for 1 week. Rats and mice were housed under controlled conditions at a temperature of 22 °C ± 2 °C, a relative humidity of 50% ± 5%, and maintained on a 12-h light/dark cycle with *ad libitum* access to standard rodent chow and water. Mice were used for evaluating anti-inflammatory and analgesic efficacy, while rats were used for toxicity evaluation. The SP group and the HP group were given the suspension prepared by mixing drug powder with 0.5% CMC-Na just before experiment. The SD group and the HD group were given the filtrate of the drug-decocted solution; the amount of water added and the decoction process were the same as the previous HPLC sample preparation.

#### 2.5.1 Mouse models of xylene-induced inflammation and formalin-induced pain

Each model, both the xylene-induced inflammation and the formalin-induced pain, consisted of 10 treatment groups (n = 8 per group), assigned in a randomized, double-blind manner: 1) control group: treated with saline, administration 0.5% CMC-Na; 2) model group: model + 0.5% CMC-Na; 3) model + SP-L group (low-dose, 0.065 g/kg, equivalent dose corresponding to the maximum daily clinical dose of HCW for an adult weighing 70 kg); 4) model + SP-H group (high-dose, 0.130 g/kg); 5) model + SD-L group (0.065 g/kg); 6) model + SD-H group (0.130 g/kg); 7) model + HP-L group (0.065 g/kg); 8) model + HP-H group (0.130 g/kg); 9) model + HD-L group (0.065 g/kg); 10) model + HD-H group (0.130 g/kg). SP and HP: 0.65 g and 1.30 g of SCW and HCW powder (40 mesh size) were weighed separately, and each was suspended in 100 mL of 0.5% CMC-Na; SD and HD: 0.65 g and 1.30 g of SCW and HCW were weighed separately, and each was soaked in water (w:v, 1:10) for 30 min. After adding water to bring to a boil, the mixture was kept simmering for another 1 h and then filtered. The filter residue was re-decocted with water (w:v, 1:10) for 30 min, and the two filtrates were combined and adjusted to a final volume of 100 mL. Mice were administered the medicinal solutions at a dose of 1 mL/100 g.

Anti-inflammatory (xylene-induced inflammation model): The ear swelling of mice induced by xylene was modeled according to the method of Jing et al. (2019) with slight modifications. Each group was treated for 7 days. 1 h after the last administration on day 7, xylene (15 μL) was smeared on both sides of the right ear of each mouse in the model group, SCW group, and HCW-treated groups, while mice of the control group were treated with saline (15 μL) as the same method. The left ear was considered as control. After 30 min, blood was collected from the retro-orbital venous plexus and centrifuged at 3,000 rpm for 15 min at 4 °C to separate the serum, which was then stored at −80 °C until analysis. The mice were then sacrificed by cervical dislocation. The levels of IL-1β, IL-6, and IL-10 in mouse serum were detected using ELISA kits (Jiangsu Kete Biotechnology Co., Ltd., Yancheng, Jiangsu) according to the manufacturer’s protocols. The left and right ears were cut along the edge of the auricle. An ear piece of the same size was punched out at the same position of the left and right ears using a puncher with a diameter of 8 mm, and then accurately weighed. The extent of ear edema was evaluated by the weight difference between the right and the left ear biopsies of the same animal. The swelling degree was calculated according to Formula 1.

$$\text{Swelling degree of the ear }\left(\%\right)=\left(\text{Right ear mass}-\text{Left ear mass}\right)/\text{Left ear mass}$$ (1)

Analgesic effect (formalin-induced pain model): For the formalin model, mice in each group were pre-placed in a transparent isolation box for environmental adaptation (2 h per day for 3 days). They were placed in an isolation box 30 min before modeling. Each group was treated for 7 days. 30 min after the last administration on day 7, 5% formalin solution (0.02 mL) was injected into the right hind toes of each mouse in the model group, SCW group, and HCW-treated groups, while mice of the control group were treated with saline (0.02 mL) as the same method. Immediately after injection, the mice were returned to the transparent isolation box. The writhing, licking, and biting behaviors directed at the injected hind paw in Phase I (0–5 min) and Phase II (15–40 min) were used as pain evaluation indices. After recording the pain reaction time of the mice, blood was collected from the retro-orbital venous plexus and centrifuged at 3,000 r/min for 15 min at 4 °C to separate the serum, which was then stored at −80 °C until analysis. The mice were then sacrificed by cervical dislocation. The levels of 5-HT and PGE_2_ in serum were detected by ELISA kits (Jiangsu Kete Biotechnology Co., Ltd., Yancheng, Jiangsu) according to the manufacturer’s protocols.

#### 2.5.2 Toxicity evaluation of SCW and HCW using Sprague-Dawley rats

Rats were selected for the toxicity study and were randomly divided into nine groups (n = 7 per group): 1) control group: 0.5% CMC-Na; 2) SP-L group (0.12 g/kg); 3) SP-H group (0.24 g/kg); 4) SD-L group (0.12 g/kg); 5) SD-H group (0.24 g/kg); 6) HP-L group (0.12 g/kg); 7) HP-H group (0.24 g/kg); 8) HD-L group (0.12 g/kg); 9) HD-H group (0.24 g/kg). SP and HP solution: 2.40 g and 1.20 g of SCW and HCW powder (40 mesh size) were weighed separately, and each was suspended in 100 mL of 0.5% CMC-Na solution. SD and HD solution: 12.00 g and 24.00 g of SCW and HCW were weighed separately, and each was soaked in water (w:v, 1:10) for 30 min. After adding water to bring to a boil, the mixture was kept simmering for another 1 h and then filtered. The filter residue was re-decocted with water (w:v, 1:10) for 30 min, and the two filtrates were combined and adjusted to a final volume of 1,000 mL. Rats were administered the medicinal solutions at a dose of 1 mL/100 g.

In preliminary experiments, rats in the SP group receiving a 0.12 g/kg dose exhibited toxic symptoms; so this dose was set as the low dose for the subsequent toxicity study. When converted to the equivalent dose for 70-kg adults, this dosage is 2.4 to 5.4 folds the clinically equivalent dose of HCW used in Mongolian medicine clinical practice (0.25–0.5 g per day). During treatment, rats were monitored daily for signs of general toxicity, including activity, fur condition, food consumption, and water intake. Body weight was recorded throughout the study, and the weight gain rate (WGR) was calculated at the endpoint (day 14). In our previous study (Zhi et al., 2020b), UPLC-MS was used for drug metabolism analysis to fully characterize the blood entry active metabolites. The methods like MS conditions, establishment of the spectrum and preparation of samples can be confirmed in the previous study.

In clinical practice, poisoning by CW can induce arrhythmias. Electrocardiogram (ECG) detection is one of the important methods for assessing cardiac toxicity. The ECG changes of the rats were detected at 15, 30, 60, 90 and 120 min after administration at three time periods respectively: after the first administration, 1 week after administration and 2 weeks after administration with the ECG-3306G electrocardiograph (Guangzhou Sanrui Electronic Technology Co., Ltd., Guangzhou, China). Before ECG collection, the rats were anaesthetized with pentobarbital sodium *via* an intraperitoneal injection, then the electrodes were inserted into the subcutaneous tissue of the rat right hind limb, left hind limb and right forelimb. The paper speed was 50 mm/s and the amplitude was 10 mm/mV. After the ECG changes were stable, the ECG data within 3 s were recorded. Heart rate (HR) was calculated according to Formula 2. The recorded ECG was quantitatively analyzed based on the Curtis and Walker (1988) arrhythmia severity scoring principle. The specific scoring method and the corresponding representative abnormal ECG during the experiment are shown in Supplementary Appendix SII.

$$\text{Heart rate }\left(\text{HR}\right)=60/0.02\times \text{The number of small grids between}\text{ two adjacent }R-\text{wave vertices on the ECG}$$ (2)

### 2.6 Biochemical analysis and histopathology

Blood biochemical analysis and histopathological examination of the heart, liver, and kidney are important methods to assist in assessing drug toxicity. After ECG recording, blood sample was collected from abdominal aorta of each anaesthetized rat. Serum was obtained by centrifuging the whole blood at 3,000 rpm for 15 min and stored at −80 °C for further biochemical investigations. These serum samples were used to assess heart, liver, and kidney function through the measurement of creatine kinase (CK), lactate dehydrogenase (LDH), N-terminal pro-brain natriuretic peptide (NT-proBNP), cardiac Troponin I (cTnI), Aspartate Aminotransferase (AST), Alanine Aminotransferase (ALT), Blood Urea Nitrogen (BUN), and Creatinine (Cr) in serum. The levels of these indices in rat serum were detected by an automatic biochemical analyzer (BK-280, Shandong Boke Biological Industry Co., Ltd., Jinan, Shandong) and ELISA test kits (Shandong Boke Biological Industry Co., Ltd., Jinan, Shandong).

At the end of the experiment, animals were sacrificed by anesthesia overdose. The heart, liver, and kidney samples were then collected to calculate the organ weight index (the ratio of organ wet weight to animal body weight, %) and perform histopathological analysis *via* HE staining. The fresh tissue was fixed in 4% paraformaldehyde solution for 24 h. After paraffin embedding, tissue sections (4 μm thick) were stained with HE staining and observed under a light microscope to evaluate histopathological changes. The level of malondialdehyde (MDA), and the activities of superoxide dismutase (SOD) and catalase (CAT) in the cardiac tissue were detected using the corresponding detection kits (Solarbio, Beijing, China) following the manufacturer’s protocol. Protein concentration was determined by BCA kit (Suzhou NCM Biotech Co., Ltd., Suzhou, Jiangsu).

### 2.7 Cell culture

The H9c2 cells were maintained in DMEM supplemented with 10% FBS, and 1% penicillin-streptomycin mixture. They were incubated at 37 °C in a humidified atmosphere containing 5% CO_2_, with the media replaced every 2–3 days. When the cells reached 80%–90% confluence, they were passaged using 0.05% trypsin for detachment.

### 2.8 Cell grouping and drug treatment

2 g each of SCW and HCW powder were weighed and extracted (power: 300 W, frequency: 40 kHz) with 50 mL methanol by ultrasonic extraction for 1 h. The extract was filtered and evaporated to dryness in 1.5 mL Eppendorf tubes under nitrogen purge. The extraction rate was calculated according to the weight change of the Eppendorf tube. Then the dried residue was dissolved in complete medium and filtered with 0.45 μm microporous membrane before use.

Initially, the 3-(4,5-Dimethylthiazol-2-yl)-2,5-diphenyltetrazolium bromide (MTT) (Lablead, Beijing, China) assay was utilized to identify the concentration range of cytotoxicity caused by SCW and HCW, which was prepared for subsequent experimental concentration selection. The following grouping and dosages were used: 1) control group: H9c2 cells were cultured without any treatment; 2) SCW group: H9c2 cells were treated with different concentrations of SP (1, 2, 5, 10, 20, 40 mg/mL); 3) HCW group: H9c2 cells were treated with different concentrations of HP (1, 2, 5, 10, 20, 40 mg/mL). Dose calculated based on the crude drug amount.

After MTT screening, the grouping and dosages were as follows: 1) Control group: H9c2 cells were cultured without any treatment; 2) SCW group: H9c2 cells were treated with different concentrations of SP (5, 10, 20 mg/mL); 3) HCW group: H9c2 cells were treated with different concentrations of HP (5, 10, 20 mg/mL).

To investigate the contribution of JNK, and p38 to SP and HP-induced cytotoxicity, H9c2 cells were pre-incubated with 10 μM of SB203580 (p38 kinase inhibitor) or 10 μM of SP600125 (JNK kinase inhibitor) for 1 h before they were treated with SP (20 mg/mL) and HP (20 mg/mL).

### 2.9 Cell viability detection

Cell viability was assessed using the MTT assay. H9c2 cells were cultured with a medium containing various concentrations of SP or HP for 24 h. Upon incubation for 24 h, MTT (0.5 mg/mL) was added to each well, followed by a 4-h incubation period inside a light-protected incubator. Subsequently, DMSO was added and the optical density (OD) was measured at 490 nm wavelength. The cell survival was calculated using the relevant Formula 3.

$$\text{Cell survival }\left(\%\right)=\text{experiment hole OD}/\text{blank hole OD}\times 100\%$$ (3)

### 2.10 Cell damage detection

Detection of LDH activity in cell culture supernatant was carried out using LDH Cytotoxicity Test Kit (Beyotime Biotechnology, Shanghai, China) following the manufacturer’s protocol.

Nucleus morphology of H9c2 cardiomyocytes was analyzed using Hoechst 33258 staining (Lablead, Beijing, China). H9c2 cells were treated with medium containing different concentrations of SP or HP for 24 h. Following the treatment, the cells were stained with Hoechst 33258 for 30 min in the absence of light. After that, they were observed under a fluorescence microscope.

The changes in the mitochondrial membrane potential of H9c2 cells were evaluated using the JC-1 dye staining method (Solarbio, Beijing, China). H9c2 cells were treated with medium containing SP or HP for 24 h. Subsequently, the H9c2 cells were treated with JC-1 staining solution and then incubated for 30 min in a 5% CO_2_ incubator at 37 °C. Cells were directly visualized with inverted fluorescence microscopy.

### 2.11 Oxidative stress and antioxidant system markers

Detection of intracellular ROS levels was carried out using DCFH-DA fluorescent probe method. H9c2 cells were treated with medium containing SP or HP for 24 h. Subsequently, the H9c2 cells were treated with medium containing DCFH-DA. Then they were incubated for 30 min in a 5% CO_2_ incubator at 37 °C. Cells were directly visualized with inverted fluorescence microscopy.

Detection of MDA, SOD, and GSH was carried out using the corresponding kit (Solarbio, Beijing, China) for testing, following the manufacturer’s protocol.

### 2.12 Western blotting (Wb)

Cells were solubilized using RIPA buffer (NCM Biotech, Suzhou, China), Protease inhibitor, and Phosphatase inhibitor (100:1:1). Equal amounts of proteins (30 µg/lane) were separated on 10% SDS-polyacrylamide gels (PAGE) and transferred to polyvinylidene difluoride (PVDF) membranes (Servicebio, Wuhan, China). These membranes were blocked with fast blocking solution (Servicebio, Wuhan, China) at RT for 10 min and then incubated with specific primary antibodies (p-p38, p-JNK, Bcl-2, and Bax, diluted 1:1,000; p38, diluted 1:2,000; JNK, diluted 1:3,000; β-Tubulin, diluted 1:5,000) at 4 °C overnight. After washing with PBS containing 1% Tween-20 (PBST) three times, membranes were then incubated with appropriate Horseradish peroxidase (HRP)-conjugated anti-rabbit (1:5,000) secondary antibody at RT for 2 h. Bound antibodies were detected with Ultra-sensitive ECL Luminescence Reagent (Yaseen Biotech, Shanghai, China) and visualized with an film developer (Bio-Rad, Hercules, CA, United States). Protein levels were quantified using ImageJ software (National Institutes of Health, Bethesda, MD, United States).

### 2.13 Statistical analysis

All statistical analyses were performed using GraphPad Prism software (version 9.5.0; San Diego, CA, United States). Data are presented as mean ± standard deviation from at least three independent experiments. For compositional data, significant differences between groups were assessed using the independent samples t-test if the data met the assumptions of normality and homogeneity of variance; otherwise, the Mann-Whitney U test was employed. For biological data, multiple comparisons were performed using one-way analysis of variance (ANOVA). If the ANOVA result was significant and the assumption of variance homogeneity was satisfied, the LSD *post hoc* test was used to compare specific groups. P-value <0.05 was considered statistically significant.

## 3 Results

### 3.1 Determination of main alkaloids in SCW and HCW

#### 3.1.1 Linearity, precision, repeatability, stability, and recovery rate

The mixed standard stock solution was diluted 1, 2, 5, 10, 25, 50, 100, and 200 folds to obtain a series of solutions to construct the linear calibration curves. The correlation coefficients (r) for the six alkaloids were calculated. The precision, repeatability, and stability of the HPLC method were evaluated for the six alkaloids in both CW powder and decoction extract. The results showed that the RSD values for precision, repeatability, and stability were all below 1.25% and 1.96%, 2.58% and 3.69%, and 2.45% and 4.39%, respectively (Supplementary Table S2). The average recoveries of the six metabolites ranged from 98.17% to 104.53% for CW powder and from 96.65% to 105.79% for decoction extract, with RSD values within 2.84% and 2.83%, respectively (Supplementary Table S3). All values were within acceptable limits, indicating that the quantitative method is accurate and reproducible.

#### 3.1.2 Processing and dosage form affected the alkaloid content in CW

**FIGURE 1:**
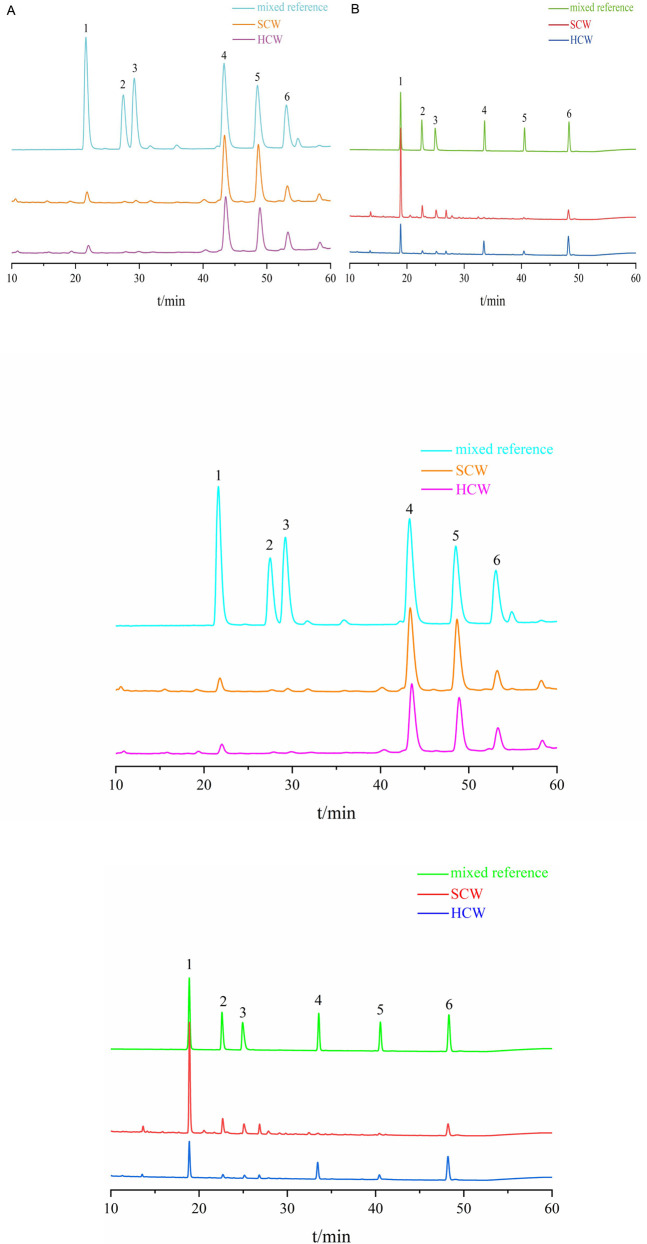
Chromatograms of SCW, HCW and mix reference standards obtained with the current HPLC system. **(A)** Determination of SCW and HCW powder samples; 1: benzoylmesaconine, 2: benzoylaconine, 3: benzoylhypaconine, 4: mesaconitine, 5: hypaconitine, 6: aconitine. **(B)** Determination of SCW and HCW decoction samples; 1: benzoylmesaconine, 2: benzoylaconine, 3: benzoylhypaconine, 4: mesaconitine, 5: aconitine, 6: hypaconitine.

The HPLC chromatograms showed good separation and a stable baseline for all six alkaloids in the different processed products of CW (Figure 1), demonstrating the suitability of the established chromatographic conditions for quantification.

The contents of the six alkaloids in various samples are summarized in Table 1. Compared with the SP, the SD exhibited a lower diester alkaloid (DA) content but a higher monoester alkaloid (MA) content. This indicates that the decoction process itself hydrolyzes highly toxic DAs into less toxic MAs. After processing the SCW with HZ decoction to obtain HCW, its powder form (HP) showed a significant reduction in the contents of both MA and DA compared with the SCW powder (SP). The DA content in HP was reduced to approximately half of that in SP. And the decrease of DA in HP did not increase the content of MA. However, substantial amounts of DA still remained in HP. Furthermore, the HD exhibited a higher total alkaloid content than its corresponding powder (HP). Compared with the SD, the HD had a higher DA content but a lower MA content.

**TABLE 1:** Quantification of main alkaloids in SCW and HCW powders and decoctions by HPLC (mg/g).

Alkaloid type	SCW		HCW	
	Powder (SP)	Decoction (SD)	Powder (HP)	Decoction (HD)
benzoylmesaconine	0.2890 ± 0.0091	3.0098 ± 0.1294	0.1924 ± 0.0040^**^	0.9744 ± 0.0212^**^
benzoylaconine	0.0280 ± 0.0002	0.4223 ± 0.0085	0.0366 ± 0.0004^*^	0.1548 ± 0.0006^**^
benzoylhypaconine	0.0608 ± 0.0008	0.5076 ± 0.0596	0.0247 ± 0.0031^**^	0.0955 ± 0.0047^*^
mesaconitine	2.4470 ± 0.0144	0.0288 ± 0.0039	1.2126 ± 0.0392^**^	0.5997 ± 0.0006^**^
hypaconitine	1.9185 ± 0.0095	0.4983 ± 0.0373	0.7463 ± 0.0253^**^	0.7937 ± 0.0735^**^
aconitine	0.5156 ± 0.0038	0.0545 ± 0.0028	0.3672 ± 0.0063^**^	0.2164 ± 0.0018^**^
Monoester alkaloids (MA)	0.3778 ± 0.0085	3.9397 ± 0.1519	0.2536 ± 0.0010^*^	1.2247 ± 0.0169^**^
Diester alkaloids (DA)	4.8812 ± 0.0212	0.5816 ± 0.0428	2.3261 ± 0.0304^**^	1.6099 ± 0.0723^**^
total alkaloids (MA+DA)	5.2607 ± 0.0266	4.5213 ± 0.1928	2.5797 ± 0.0310^**^	2.8346 ± 0.0585^**^

Abbreviations: MA, the sum of benzoylmesaconine, benzoylaconine, and benzoylhypaconine; DA, the sum of mesaconitine, hypaconitine, and aconitine.

^**^ *p* < 0.01.

^*^ *p* < 0.05: SP, vs HP, SD, vs HD.

### 3.2 Anti-inflammatory and analgesic effects of SCW and HCW

**FIGURE 2:**
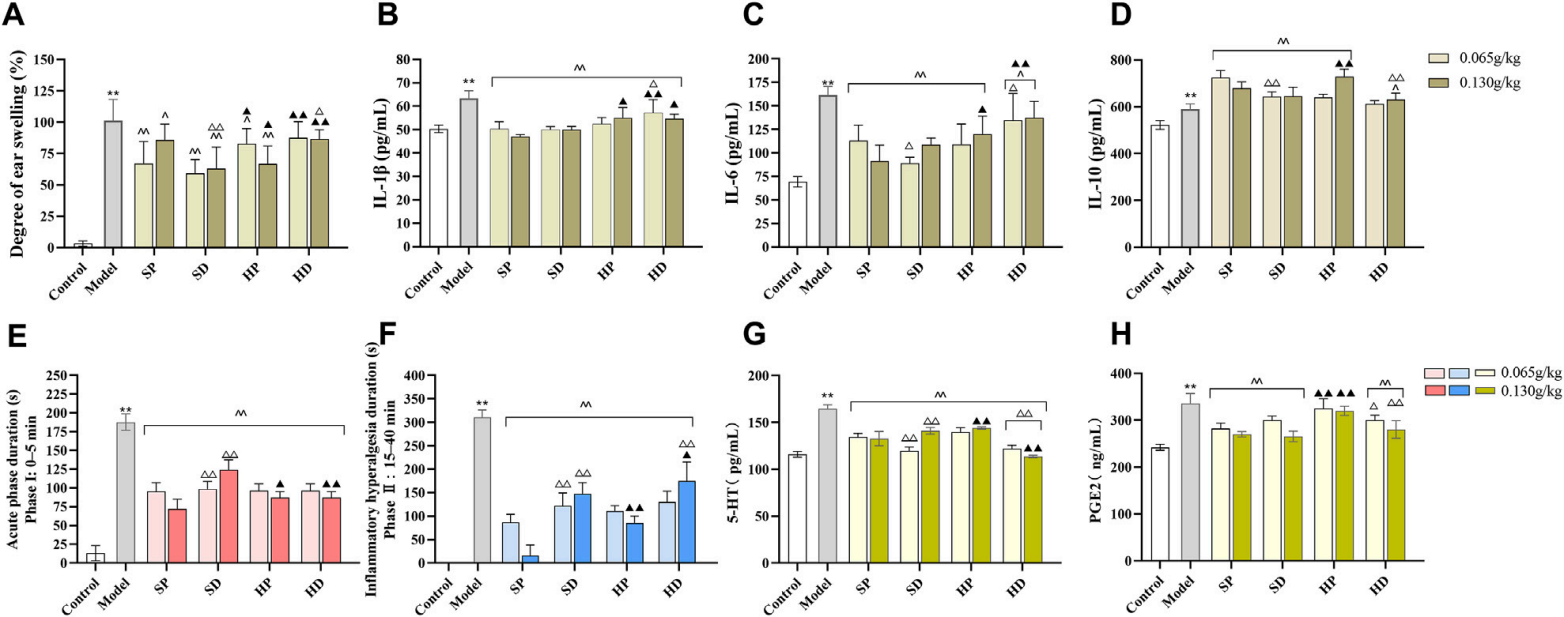
Anti-inflammatory and analgesic effects of SCW and HCW in xylene-induced ear edema and formalin-induced pain models. After 7 days of administration (low/high doses) of SCW/HCW (powder: SP, HP; decoction: SD, HD), models were established after the final administration. **(A–D)** Xylene was applied to the right ear to induce edema, ear swelling was measured, and inflammatory factors (IL-1β, IL-6, and IL-10) in serum were detected using an ELISA kit. **(E–H)** After formalin injection into the right hind paw, the total duration of pain response in the 0–5 min and 5–40 min was recorded, and serum pain-related factors (5-HT and PGE_2_) were detected by ELISA kit (*x* ± *s*, *n* = 8. Compare to control, ^**^ *p* < 0.01; compare to model, ^^^^ *p* < 0.01, ^^^ *p* < 0.05; at the same dose, SP vs SD, HP vs HD, ^△△^ *p* < 0.01, ^△^ *p* < 0.05; at the same dose, SP vs HP, SD vs HD, ^▲▲^ *p* < 0.01, ^▲^ *p* < 0.05).

Results from the mouse ear swelling experiment showed that the ear swelling of the model group was significantly increased compared with that of the control group (^**^ *p* < 0.01), indicating successful model establishment. Compared with the Model group, both SCW and HCW (in decoction or powder form) reduced ear swelling and exerted anti-inflammatory effects (Figure 2A), with significant differences observed in the SP, SD, and HP groups (^ ^*p* < 0.01, ^*^*^ *p* < 0.05). Although HD showed a tendency towards anti-inflammatory activity, the difference compared with the Model group did not reach statistical significance. Notably, the SD group not only reduced the content of toxic DAs but also showed a greater reduction in ear swelling compared with the SP group (SP-H vs SD-H, ^△△^ *p* < 0.01). Furthermore, the ear swelling degree in the HP-H group was significantly lower than that in the HD-H group (^△^ *p* < 0.05) and the SP-H group (^▲^ *p* < 0.05). The results of inflammatory factor levels showed that IL-1β, IL-6, and IL-10 in the model group were significantly increased compared with the control group (^**^ *p* < 0.01). Compared with the model group, IL-1β was decreased in SP, SD, HP and HD group (^ ^*p* < 0.01), IL-6 was decreased in SP, SD, HP and HD group (^ ^*p* < 0.01, ^*^*^ *p* < 0.05), while IL-10 was increased in SP, SD, HP and HD-H group (^ ^*p* < 0.01, ^*^*^ *p* < 0.05) (Figures 2B–D).

Similarly, compared with the control group, the formalin pain behavior duration of mice in the model group was significantly increased (^**^ *p* < 0.01), indicating that the formalin-induced pain model was successful. The duration of pain response in the SP, SD, HP, and HD administration groups was significantly shorter than that in the model group (^ ^ *p* < 0.01), indicating that SP, SD, HP, and HD could relieve pain (Figures 2E,F). During the phase I acute pain period, the analgesic effects of different groups were ranked as follows: SP > HP > HD > SD. During the phase II inflammatory pain period, the analgesic effects were: SP > HP > SD > HD. Furthermore, compared with the control group, the contents of 5-HT and PGE_2_ in the serum of mice in the model group increased (^**^ *p* < 0.01) (Figures 2G,H). After administration, compared with the model group, the contents of 5-HT in the serum of mice in each administration group were significantly lower (^ ^ *p* < 0.01). Except for the HP group, the contents of PGE_2_ in the serum of mice in the other groups decreased significantly compared with those in the model group (^ ^*p* < 0.01).

### 3.3 Toxic effects of SCW and HCW *in vitro*

#### 3.3.1 Effects of SCW and HCW on general toxicity in rats

**FIGURE 3:**
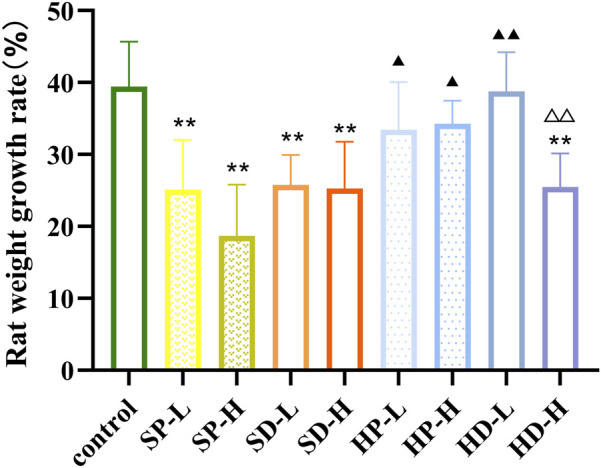
Weight growth rate of rats after 14 days of low (0.12 g/kg) or high (0.24 g/kg) doses of SCW, HCW powder (SP, HP) and decoction (SD, HD) continuous administration (*x* ± *s, n* = 7. Compared with control, ^**^ *P* < 0.01; at the same dose, SP vs SD, HP vs HD, ^△△^ *p* < 0.01; at the same dose, SP vs HP, SD vs HD, ^▲▲^ *p* < 0.01, ^▲^ *p* < 0.05).

Rats in the control group appeared alert and active. Their eating, drinking and defecation behaviors were normal; they responded quickly to external stimuli, and their fur was smooth and glossy. In contrast, rats in the SCW group appeared lethargic and inactive. Their fur appeared ruffled and unkempt, locomotor activity was markedly reduced, and consumption of food and water was decreased. Following each administration of SCW, some rats adopted a curled posture and exhibited a significant increase in respiratory amplitude. Rats in the SD group and the HD-H group also showed similar but milder adverse effects, including reduced activity, poor fur condition, and a slight decrease in food and water intake. The general condition and activity levels of rats in the other administration groups remained comparable to those of the control group. Accordingly, we calculated the WGR of each group of rats after 14 days of administration (Figure 3). Compared with the control group, the WGR of rats in the SP, SD, and HD-H groups was significantly decreased (^**^ *p* < 0.01), indicating that the WGR of the rats was inhibited. Compared with the SCW groups (SP and SD), the WGR was higher in most HCW groups (except for the HD-H group), suggesting that HCW had a less adverse impact on weight gain than SCW and that processing reduced the toxicity of SCW. In addition, there were significant differences in the WGR of rats between the HP-H group and the HD-H group (^△△^ *p* < 0.01). These results were consistent with the observed general condition of the rats.

#### 3.3.2 Types of arrhythmia caused by SCW and HCW in rat ECG

**FIGURE 4:**
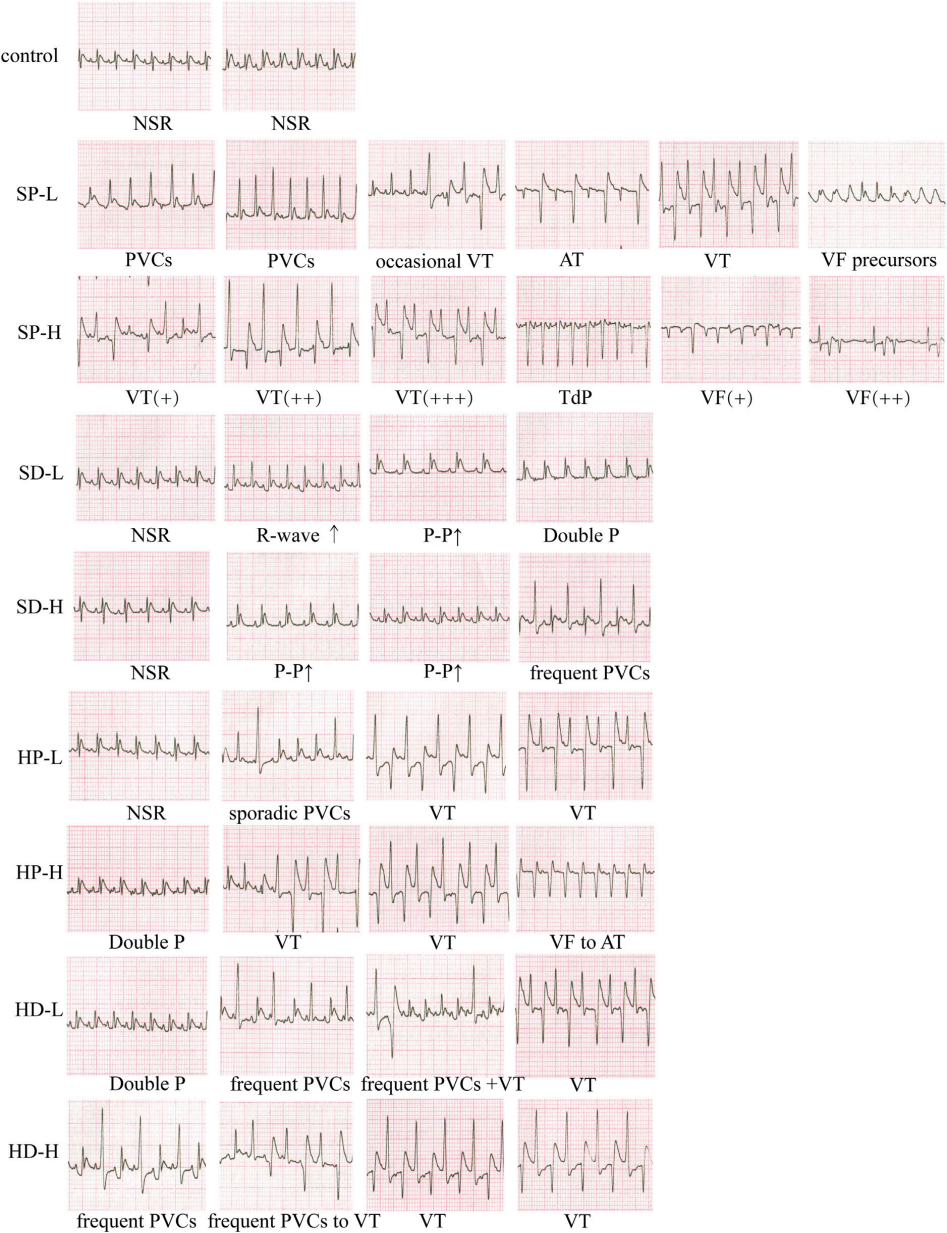
Effect of each group on the electrocardiogram (ECG) of rats. Rats received low or high doses of SCW or HCW (powder: SP/HP; or decoction: SD/HD) for 14 days. Recordings were taken at multiple timepoints post-administration. NSR, normal sinus rhythm; AT, atrial tachycardia; PVCs, premature ventricular complexes; VT, ventricular tachycardia; VF, ventricular fibrillation; TdP, Torsades de Pointes. Symbols, +, severity; ↑, interval prolongation.

Representative ECGs from each group during the treatment week were analyzed to classify arrhythmia types (Figure 4). ECGs from control group rats exhibited normal P waves and QRS complexes, consistent with normal sinus rhythm (NSR). In contrast, ECGs from the SP-L group revealed various arrhythmias, including premature ventricular complexes (PVCs), occasional ventricular tachycardia (VT), atrial tachycardia (AT), and precursors to ventricular fibrillation (VF). Administration of SP-H induced more severe arrhythmias, characterized by VT, Torsades de Pointes (TdP), and the disappearance of normal P-QRS-T waves with irregular continuous waveforms indicative of VF. Analysis of the SD-L group revealed a spectrum of ECG findings. These included ECGs exhibiting NSR, others showing augmented R-wave amplitude, some with reduced heart rate indicated by prolonged P-P intervals, and double P waves characteristic of atrial abnormality. For the SD-H group, while some ECGs also displayed NSR, others showed decreased HR. Additionally, frequent PVCs were present. Representative ECGs from the HP-L group showed NSR (with a slightly augmented Q-wave amplitude), sporadic PVCs, and VT. In the HP-H group, ECGs revealed atrial arrhythmia with double P waves, VT, and recovery from VF to AT. For the HD-L group, observed arrhythmias were atrial arrhythmia (double P waves), frequent PVCs, and VT. As for the HD-H group, the ECGs revealed frequent PVCs and VT, with P-wave inversion or disappearance observed during VT.

#### 3.3.3 Effects of SCW and HCW on ECG heart rate (HR) and arrhythmia score in rats

**FIGURE 5:**
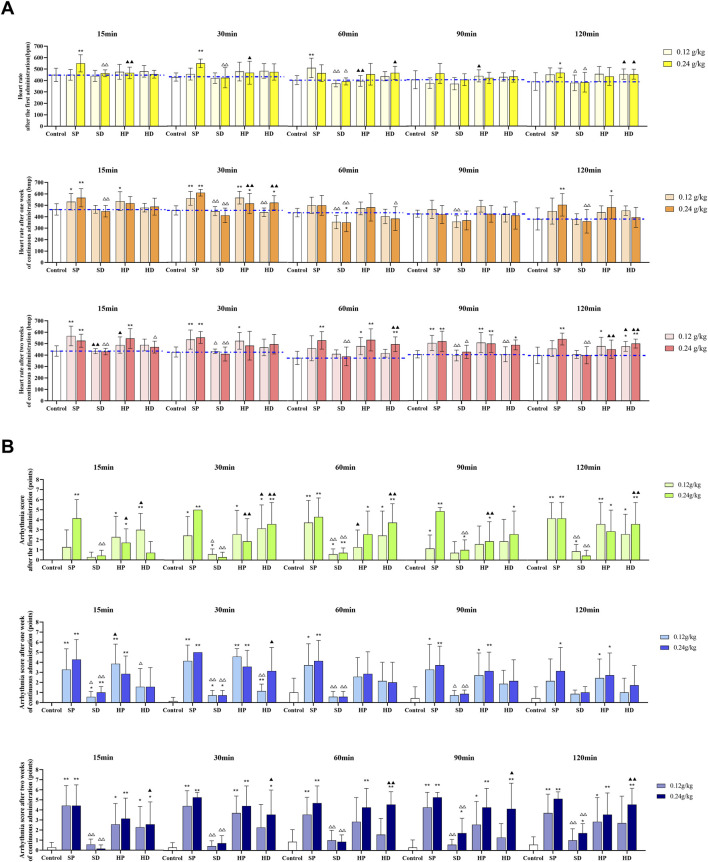
Effects of different samples of CW on electrocardiogram (heart rate and arrhythmia score) in rats. Low (0.12 g/kg) and high (0.24 g/kg) doses of SCW, HCW powder (SP, HP) and decoction (SD, HD) were administered continuously for 14 days. The electrocardiogram was recorded at 15, 30, 60, 90, and 120 min after administration on days 0, 7 and 14. **(A)** Heart rate. **(B)** Arrhythmia quantitative score (*x* ± *s, n* = 7. Compared with control, ^**^ *p* < 0.01, ^*^ *p* < 0.05; at the same dose, SP vs SD, HP vs HD, ^△△^ *p* < 0.01, ^△^ *p* < 0.05; at the same dose, SP vs HP, SD vs HD, ^▲▲^ *p* < 0.01, ^▲^ *p* < 0.05).

According to the type of arrhythmia determined by each group, HR (Figure 5A) and arrhythmia score (Figure 5B) were used to comprehensively evaluate the ECG of each group of rats. After the first administration, compared with the control group, the SP group showed a significantly increased HR. Specifically, the SP-H group exhibited a markedly increased HR at 15 and 30 min (^**^ *p* < 0.01), while the SP-L group showed a significant increase at 60 min (^**^ *p* < 0.01). By 90 min, the HR in the SP-L group had returned to normal levels. The SD group showed no significant difference in HR compared with the control group. However, the HR of the SD group was significantly lower than that of the SP group. This difference was particularly evident in the high-dose groups, where the SD-H group had significantly lower HR values at 15 and 30 min compared with the SP-H group (^△△^ *p* < 0.01). Neither the HP nor the HD groups altered HR significantly compared with the control group. However, when compared with the SP groups, the HP groups showed reduced HR: the HP-H group had a significantly lower HR at 15 (^▲▲^ *p* < 0.01) and 30 min (^▲^ *p* < 0.05), and the HP-L group showed a significant reduction at 60 min (^▲▲^ *p* < 0.01). Conversely, a comparison between the decoction groups revealed an opposite trend to that seen between the powder groups. The HR of the HD group was higher than that of the SD group. In the low-dose groups, this difference was statistically significant at 60 and 120 min (^▲^ *p* < 0.05).

Following continuous administration for 1 week, compared with the control group, the SP-L group showed increased HR at 15 min (^*^ *p* < 0.05) and 30 min (^**^ *p* < 0.01), while the SP-H group demonstrated significantly increased HR at 15, 30, and 120 min (^**^ *p* < 0.01). Conversely, the SD group showed a tendency to decrease HR, which became statistically significant at 60 min (^**^ *p* < 0.01). In addition, comparing the SD group to the SP group, the SD-L group showed markedly reduced HR at 30, 60, and 90 min (^△△^ *p* < 0.01) *versus* the SP-L group. The SD-H had significantly lower HR at 15, 30, 60, and 120 min (^△△^ *p* < 0.01) *versus* the SP-H group. Furthermore, compared with the control group, the HP-L group showed increased HR at 15 min (^*^ *p* < 0.05) and 30 min (^**^ *p* < 0.01), and the HP-H group showed increases at 30 and 120 min (^*^ *p* < 0.05). The HR in the HP group was largely similar to that in the SP group. However, the HD group displayed higher HR values than the SD group, which was significant for the HD-H group at 30 min (^▲▲^ *p* < 0.01). Furthermore, the HD group had a lower HR than the HP group, with significant differences observed in the low-dose groups at 30 min (^△△^ *p* < 0.01), and in the high-dose groups at 60 min (^△^ *p* < 0.05).

After 2 weeks of continuous administration, the heart rates of rats in the SP and HP groups were still significantly increased compared with the control group, consistent with the trend observed after 1 week of administration. Interestingly, for the HD group, with the exception of one monitoring time point (HD-L group, at 90 min), the HR at all other time points was higher than that in the control group, and the HR at some time points showed a significant increase compared with the control group (at 60, 90 and 120 min). This change differs from that of the HD group after the first administration (the HD group showed no significant difference in HR compared with the control group). Notably, the HR in the SD group remained stable and close to that of the control group throughout the administration period, showing no significant changes.

From the arrhythmia scores of each group (Figure 5B), it can be seen that the SP group was the most arrhythmogenic, showing significantly higher scores than all other groups. In contrast, the arrhythmia scores of the SD group showed no significant difference compared with the control group. In the SP-H group, the arrhythmia scores remained at a relatively high level throughout the administration period. And in the SP-L group, the arrhythmia scores showed an upward trend as the administration time increased. For HCW, both the HP and HD groups produced relatively high arrhythmia scores. The HP group maintained its arrhythmia scores within a stable range over the entire administration period, whereas the HD group showed a dynamic change: its arrhythmia scores were comparable to those of the HP group after 2 weeks of continuous administration.

#### 3.3.4 Toxic effects of SCW and HCW on heart, liver, and kidneys in rats

**FIGURE 6:**
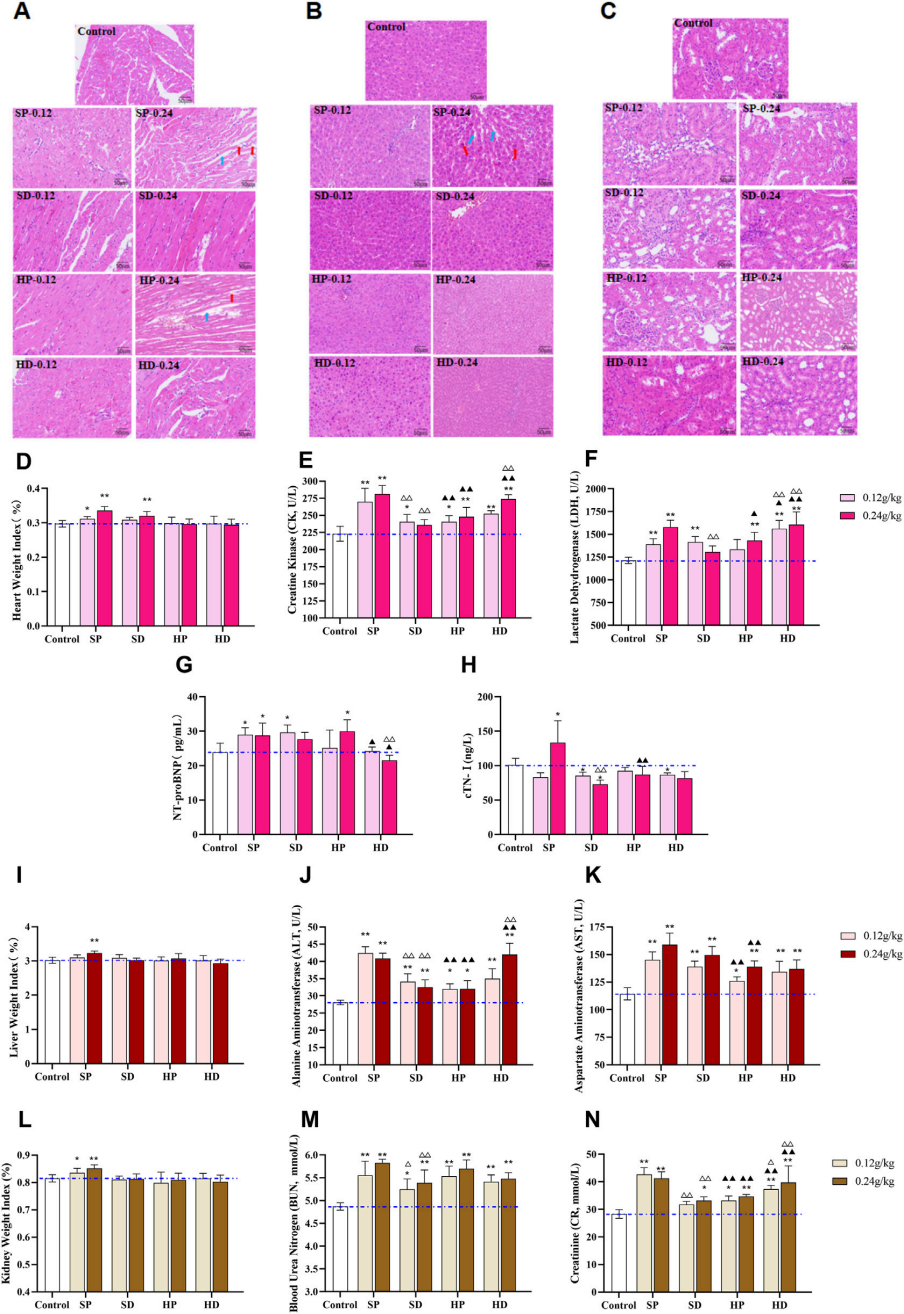
Effects of different samples of CW on heart, liver and kidney of rats after 14 days of continuous administration. **(A–C)** Rats were continuously administered with low (0.12 g/kg) and high (0.24 g/kg) doses of SCW, HCW powder (SP, HP) and decoction (SD, HD) for 14 days, and distinct effects on histopathology of heart **(A)**, liver **(B)** and kidney **(C)** were observed *via* hematoxylin-eosin (HE) staining (×200). **(A)** H&E staining of heart tissue in the SP-H and HP-H groups showed an increased myocardial interstitial space (blue arrows) and breakage and dissolution of cardiomyocyte fibers (red arrows). **(B)** H&E staining of liver tissue in the SP-H group showed hepatocyte enlargement (red arrows) and increased space between hepatic cords (blue arrows). **(D)** Cardiac weight index. **(E–H)** Cardiac function-related serum biomarkers: CK, LDH, NT-proBNP, cTN-I. **(I)** Liver weight index. **(J and K)** Liver function-related serum biomarkers: ALT and AST. **(L)** Kidney weight index. **(M and N)** Kidney function-related serum biomarkers: BUN and Cr. (*x* ± *s, n* = 7. Compared with control, ^**^ *p* < 0.01, ^*^ *p* < 0.05; at the same dose, SP vs SD, HP vs HD, ^△△^ *p* < 0.01, ^△^ *p* < 0.05; at the same dose, SP vs HP, SD vs HD, ^▲▲^ *p* < 0.01, ^▲^ *p* < 0.05).

Histopathological and serum biomarkers further provided a basis for the difference in toxicity between the SCW group and the HCW group. Specifically, SP administration resulted in significantly elevated cardiac weight index (Figure 6D), increased serum CK and LDH levels (Figures 6E,F), and histopathological damage including disorganized myocardial fiber alignment, interstitial edema, and fiber rupture (Figure 6A). In contrast, the SD markedly attenuated these toxic effects, with normal cardiac histological structure and reduced CK and LDH levels. Notably, the HP partially mitigated cardiotoxicity (lower cardiac weight index, CK and LDH levels vs SP), yet the HP-H group still caused loose myocardial fiber arrangement. The HD further improved histopathology compared with the HP, but serum CK and LDH were still at a high level. To further confirm myocardial damage and heart failure, we measured NT-proBNP and cTnI. SP significantly elevated NT-proBNP and cTnI levels, confirming its relatively high cardiotoxicity (Figures 6G,H). In the HP-H group, the content of NT-proBNP increased, suggesting potential safety risks associated with high-dose HP administration.

In addition, 2 weeks after administration, the liver and kidney weight indexes of rats in the SP-H group were significantly increased (^**^ *p* < 0.01), suggesting that the SP might induce substantial lesions in the liver and kidneys of rats (Figures 6I,L). Serum biochemistry analysis revealed elevated levels of ALT, AST, BUN, and Cr in several groups, with the most pronounced increases observed in the SP-H group. This indicated that SCW and HCW could cause abnormal liver and kidney function to varying degrees (Figures 6J–N). Pathological examination showed that only the rats in the high-dose SP group had liver damage, while there was no obvious liver and kidney damage in the rats of other groups, which might be because the damage did not reach the threshold of histological lesions (Figures 6B,C). Except for BUN, the levels of AST, ALT, and Cr in the HD were higher than those in the HP group, indicating that the HD exerted greater toxicity to the liver and kidneys to a certain extent.

#### 3.3.5 Effects of SCW and HCW on oxidative stress-related indicators in rat myocardial tissue

**FIGURE 7:**
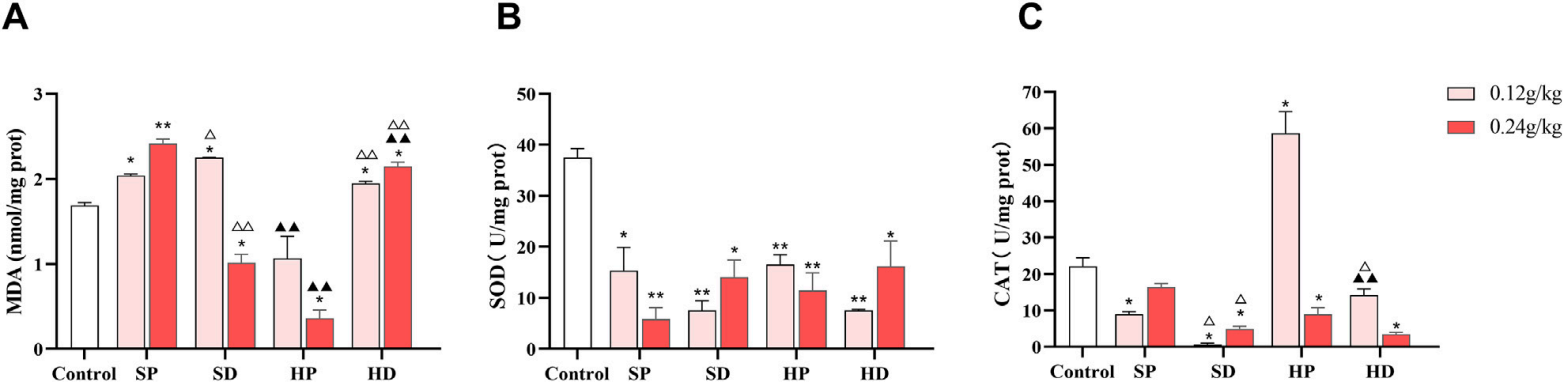
Effect of low (0.12 g/kg) and high doses (0.24 g/kg) of SCW/HCW (powder: SP/HP; decoction: SD/HD) on rat myocardial oxidative damage. **(A–C)** MDA level, SOD activity, and CAT activity in rat myocardial tissue were measured by ELISA (*x* ± *s, n* = 3. Compared with control, ^**^ *p* < 0.01, ^*^ *p* < 0.05; at the same dose, SP vs SD, HP vs HD, ^△△^ *p* < 0.01, ^△^ *p* < 0.05; at the same dose, SP vs HP, SD vs HD, ^▲▲^ *p* < 0.01).

Since myocardial injury is often accompanied by an imbalance in oxidative stress, we measured the content of MDA and activities of SOD and CAT in the myocardial tissues of rats. Compared with the control group, in the myocardial tissues of rats in the SCW intervention groups, except for the high-dose group of the SD, the rest of the groups exhibited the characteristics of increased MDA content and decreased SOD and CAT activities (Figures 7A–C). This result indicated that SCW can induce oxidative stress damage in the hearts of rats, and there were differences in the process and degree of damage between the SP and the SD group, suggesting that different dosage forms may affect the degree of the cardiotoxic effect of SCW. In addition, the HP group showed a certain antioxidant effect. The content of MDA in myocardial tissue of rats in HP-H group was significantly lower than that in control group (^*^ *p* < 0.05), and HP-L and HP-H were significantly lower than that in SP corresponding dose group (^▲▲^ *p* < 0.01), and the CAT activities in the HP-L group were significantly higher. This result indicated that after being processed with HZ decoction, the cardiotoxicity of SCW was weakened, the antioxidant capacity was enhanced, and the level of oxidative damage to the hearts of rats could be reduced, especially when administered at a low dose. It is worth noting that, compared with the HP group, the MDA content in the myocardial tissues of the HD group was higher, and the CAT activities were lower. Furthermore, the MDA content in the HD group was higher than that in the control group (^*^ *p* < 0.05), indicating that the protective effect of the HD group on cardiac oxidative damage was weaker than that of the HP group, and even exacerbated the oxidative stress damage to the heart to a certain extent.

### 3.4 Toxic effects of SP and HP on H9c2 cardiomyocytes

#### 3.4.1 Effect of SP and HP on H9c2 cell viability

**FIGURE 8:**
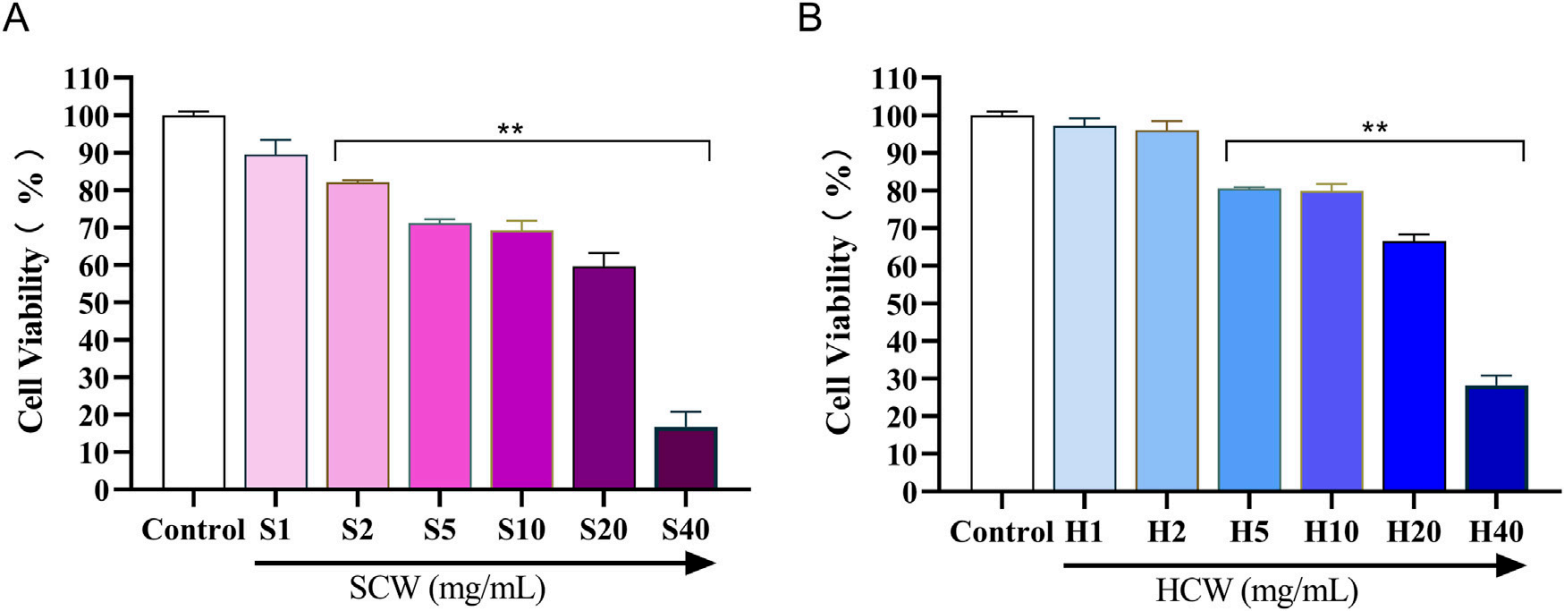
Viability of H9c2 cardiomyocytes treated with different concentrations of SCW powder (SP) and HCW powder (HP), determined by the MTT assay. **(A)** Effect of SP on cell viability. **(B)** Effect of HP on cell viability (*x* ± *s, n* = 3. Compared with control, ^**^ *p* < 0.01).

As shown in Figure 8, the cell survival rate decreased when the concentration of SP was in the range of 2 mg/mL to 40 mg/mL and that of HP was in the range of 5 mg/mL to 40 mg/mL (^**^ *p* < 0.01). In order to further compare the toxicity of SP and HP to cardiomyocytes, 5, 10, and 20 mg/mL were selected for both SP and HP for subsequent experiments.

#### 3.4.2 Effect of SP and HP on H9c2 LDH release

**FIGURE 9:**
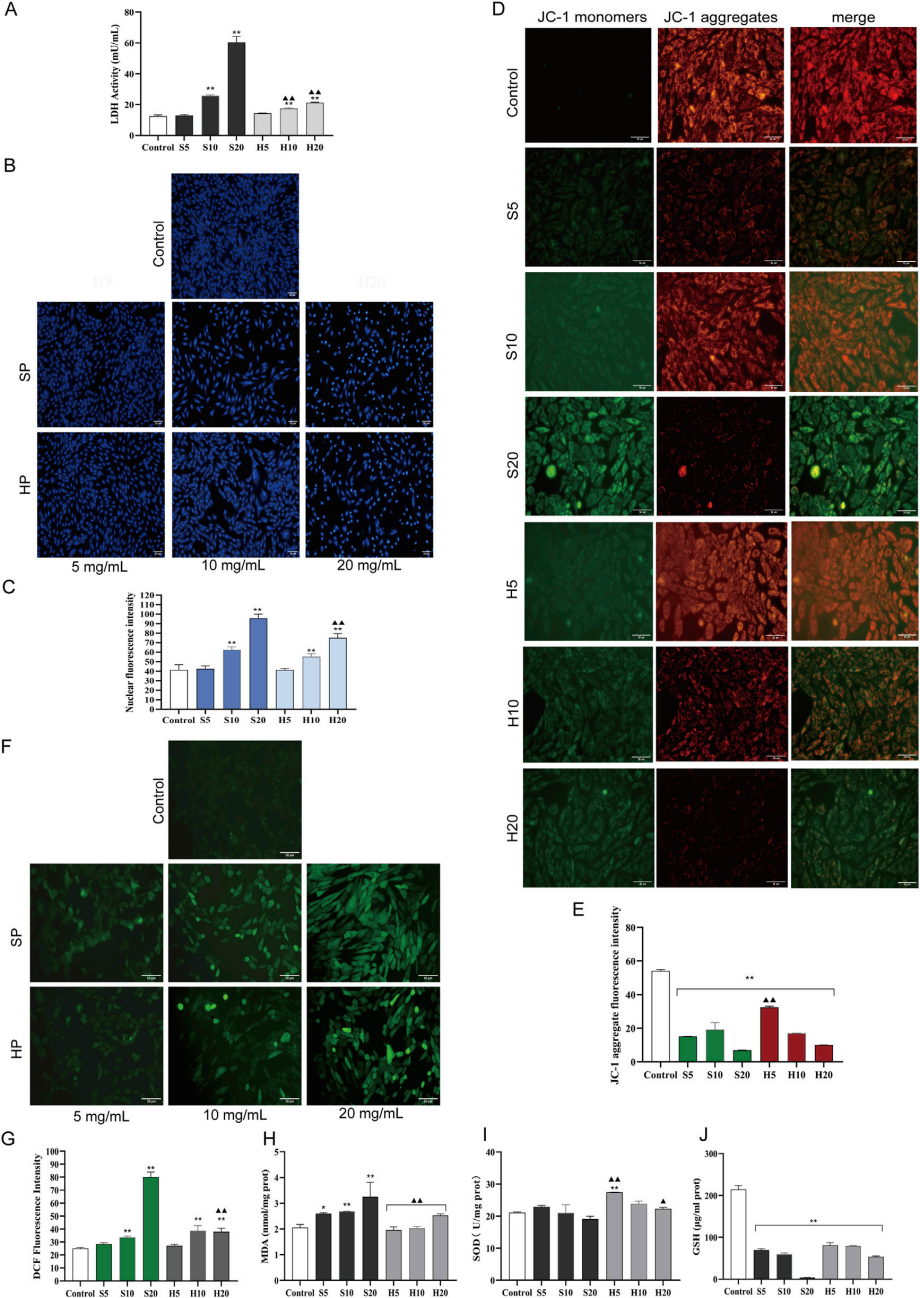
The toxic effect of HCW on H9c2 cardiomyocytes was weaker than that of SCW. These cells were treated with SCW/HCW powder (SP/HP) at 5, 10 or 20 mg/mL. **(A)** LDH activity in the culture supernatant of H9c2 cells by ELISA. **(B)** Effect of SCW or HCW on cell nucleus apoptosis in H9c2 cells by Hoechst 33258 staining, fluorography (×100). **(C)** Quantification is expressed as the mean fluorescence intensity. **(D)** Effect of SCW or HCW on mitochondrial membrane potential in H9c2 cells by JC-1 dye staining, fluorography (×100). **(E)** Quantification is expressed as the JC-1 aggregate fluorescence intensity. **(F)** Effect of SCW or HCW on ROS release in H9c2 cells by DCFH-DA, fluorography (×100). **(G)** Quantification is expressed as the DCF fluorescence intensity. **(H–J)** Detection of MDA, SOD, and GSH in H9c2 cells by ELISA (*x* ± *s, n* = 3. Compared with control, ^**^ *p* < 0.01, ^*^ *p* < 0.05; at the same dose, SP vs HP, SD vs HD, ^▲▲^ *p* < 0.01, ^▲^ *p* < 0.05).

Compared with the control group, a significant increase in LDH release was observed in all groups except S5 and H5 (^**^ *p* < 0.01). Among them, the S20 group showed the highest LDH release (Figure 9A). Furthermore, LDH release was significantly reduced in the H10 and H20 groups compared with the S10 and S20 groups, respectively (^▲▲^ *p* < 0.01).

#### 3.4.3 Effects of SP and HP on the nuclear in H9c2 cardiomyocyte

Nuclear changes in cells were obtained by Hoechst 33258 staining (Figures 9B,C). Hoechst 33258 specifically stains the nucleus, and when the cell becomes apoptotic, its nuclei are colored blue and fragmented. In the control group, the nuclei displayed light blue fluorescence after Hoechst 33258 staining, and their shapes remained unaltered. Compared with the control group, with the increase of the dose, the SP and HP groups showed the characteristics of nuclear apoptosis, especially in the S20 and H20 groups (^**^ *p* < 0.01). The nuclear chromatin was significantly condensed, and some fragments were observed. On the whole, cells in the S20 group were significantly sparser, but the fluorescence showed dense and strong bright blue signals. Notably, the degree of nuclear pyknosis in the H20 group was less severe than that in the S20 group. Consistent with this morphological observation, the average fluorescence intensity of the H20 group was significantly lower than that of the S20 group (^▲▲^ *p* < 0.01), indicating relatively weaker apoptotic signals.

#### 3.4.4 Effect of SP and HP on the mitochondrial membrane potential in H9c2 cardiomyocyte

Changes in mitochondrial membrane potential, an early indicator of apoptosis, were measured using JC-1 staining (Figures 9D,E). In healthy cells with high mitochondrial membrane potential, JC-1 forms aggregates that emit red fluorescence, whereas in apoptotic cells with low mitochondrial membrane potential, it remains as monomers that emit green fluorescence. In the control group, predominantly orange-red fluorescence was observed, with only a small amount of green fluorescence, indicating higher mitochondrial membrane potential. Stronger green fluorescence and weaker orange-red fluorescence were observed in the S20 and H20 groups (^**^ *p* < 0.01), demonstrating a significant decline in mitochondrial membrane potential. Compared with the control group, the average fluorescence intensity of JC-1 aggregates (red fluorescence) in each experimental group was significantly decreased (^**^ *p* < 0.01). Among them, the red fluorescence intensity of the S20 group was the lowest, indicating that the S20 group had obvious cardiotoxicity to myocardial cells, leading to cell apoptosis. When comparing the HP groups and SP groups at the same dose, it was found that the average red fluorescence intensity of the H5 group was significantly higher than that of the S5 group (^▲▲^ *p* < 0.01). This higher red fluorescence intensity indicates an increase in mitochondrial membrane potential, which suggests a reduction in the toxic effects of HP (H5) compared with SP (S5).

#### 3.4.5 Effects of SP and HP on oxidative stress-related indicators in H9c2 cardiomyocytes

Abnormal activation of apoptosis is often accompanied by an imbalance of redox homeostasis. The control group, in which intracellular ROS levels in H9c2 cardiomyocytes were detected *via* the DCFH-DA fluorescent probe, exhibited only weak green fluorescence (indicating low basal ROS production), whereas significantly elevated intracellular ROS levels were observed in the middle- and high-dose groups of both SP and HP compared to the control group (^**^ *p* < 0.01; Figures 9F,G). Among the treatment groups, the S20 group showed the highest average fluorescence intensity, indicating the most severe ROS accumulation (^**^ *p* < 0.01). Biochemical assays showed that the MDA content increased, while SOD activity and GSH content decreased in the S20 group, indicating that SCW would produce a large amount of oxygen free radicals at high doses, consume antioxidant enzymes, and induce oxidative stress in cardiomyocytes. Compared with S20, the average fluorescence intensity of H20 group was significantly decreased (^▲▲^ *p* < 0.01), the release of ROS was decreased, the content of MDA was decreased, the activities of SOD, and the content of GSH were increased, indicating that the degree of oxidative damage of the cells in the HP group was lower than that in the SP group.

### 3.5 Mechanism of SP and HP leading to the toxicity of H9c2 cardiomyocytes through p38/JNK MAPK signaling pathway

#### 3.5.1 SP and HP influences p38/JNK MAPK signaling pathways

**FIGURE 10:**
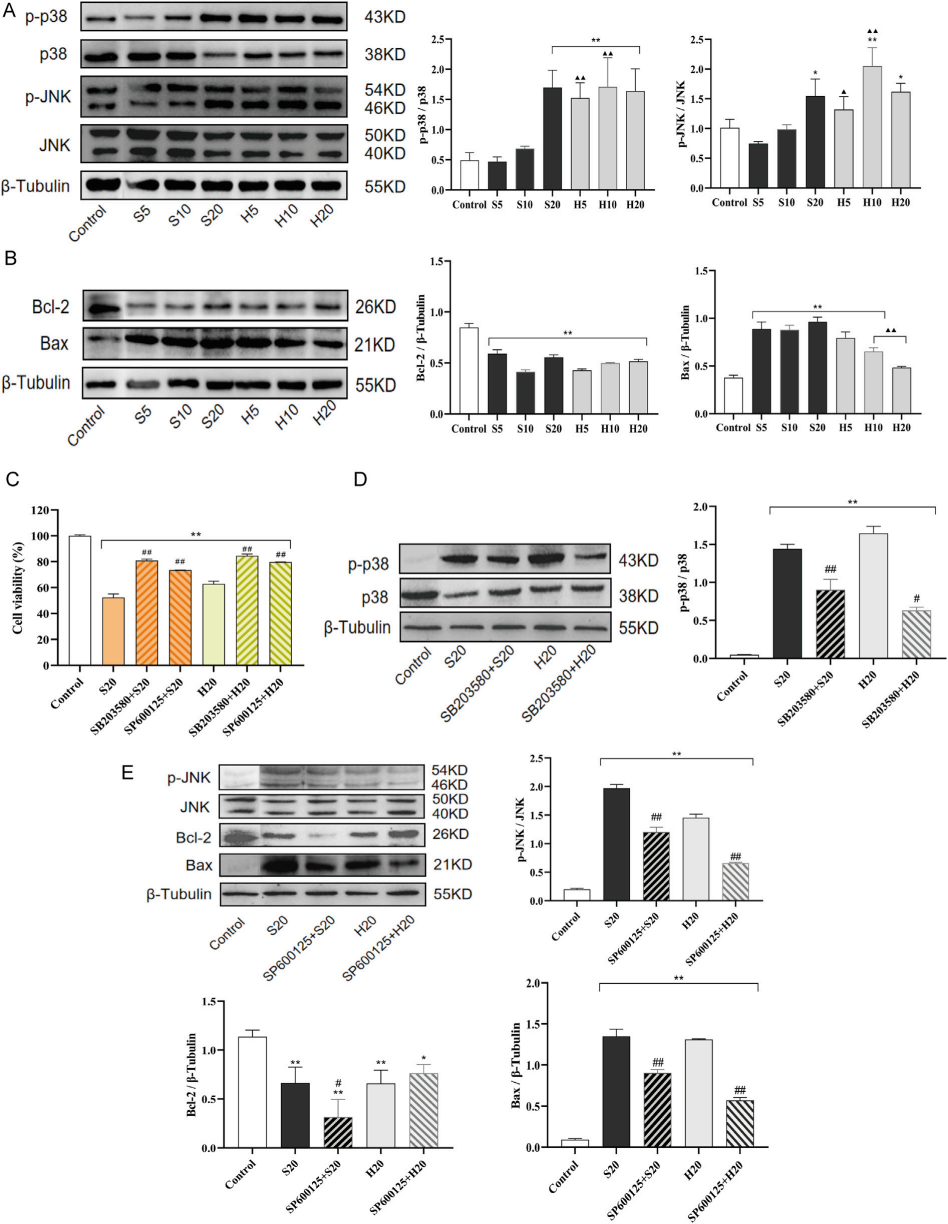
Cardiomyocyte toxicity induced by SCW and HCW *via* activation of the p38/JNK pathway and mitochondrial apoptosis. Effect of SCW and HCW on the expression of Phosphorylated p38, Phosphorylated JNK, Bcl-2, and Bax in H9c2 Cells Detected by Western Blot (Wb). **(A)** Wb analysis of p-p38/p38 and p-JNK/JNK expression levels and quantitation of Wb band intensity in H9c2 cells. **(B)** Wb analysis of Bcl-2 and Bax expression levels and quantitation of Wb band intensity in H9c2 cells. These cells were treated with SCW/HCW powder (SP, HP) at 5, 10 or 20 mg/mL. **(C)** Cells were pretreated with p38 (SB203580) and JNK (SP600125) inhibitor for 1 h and then treated with 100 μL 20 mg/mL SCW or HCW powder for 24 h. Graphs demonstrate changes in cell viability in H9c2 cells as assessed by the MTT assay. **(D and E)** Cells were pretreated with p38 (SB203580) and JNK (SP600125) inhibitor for 1 h and then treated with 100 μL 20 mg/mL SCW or HCW powder for 24 h. Wb analysis and quantitation of p-p38/p38, p-JNK/JNK, Bcl-2, and Bax expression in H9c2 cells with or without p38/JNK inhibition (*x* ± *s, n* = 3. Compared with control, ^**^ *p* < 0.01, ^*^ *p* < 0.05; at the same dose, SP vs HP, ^▲▲^ *p* < 0.01, ^▲^ *p* < 0.05; comparison between groups with and without p38/JNK inhibition, ^*##*^ *p* < 0.01, ^*#*^ *p* < 0.05).

To determine whether SP/HP induce cardiomyocyte toxicity by modulating p38/JNK MAPK signaling pathways, Wb analysis was performed on SP- or HP-treated H9c2 cells. As shown in Figures 10A,B, phosphorylation levels of p38 and JNK were upregulated by SP or HP treatment, and the change was significant in the high-dose group (S20 and H20) (^**^ *p* < 0.01). In addition, given the role of p38/JNK MAPK signaling in regulating the mitochondrial apoptosis pathway, the expression of pro-apoptotic and anti-apoptotic Bcl-2 proteins was examined. As shown in Figure 10B, the expression level of the anti-apoptotic protein Bcl-2 was significantly decreased in all treatment groups compared to the control, whereas that of the pro-apoptotic protein Bax was increased. Compared with the SP10 and SP20 groups, the expression level of Bax in H10 and H20 groups was significantly decreased (^▲▲^ *p* < 0.01). While the reduction of anti-apoptotic Bcl-2 was comparable between SP and HP groups, Bax expression was significantly lower in the H10 and H20 groups compared to the S10 and S20 groups.

To explore the role of p38/JNK MAPK activation in SP or HP-triggered cell injury, inhibitor experiments were conducted. Pretreatment with SB203580 or SP600125 for 1 h not only prevented the decrease in cell viability caused by S20 and H20 treatment (Figure 10C), but also significantly inhibited the S20- and H20-induced phosphorylation of p38 and JNK (Figures 10D,E). The JNK inhibitor SP600125 elicited contrasting effects on Bcl-2 expression in SP *versus* HP-treated cells. While SP600125 pretreatment did not rescue the suppressed Bcl-2 expression in the S20 group, it caused a non-significant increasing trend in Bcl-2 expression in the H20 group compared to H20 treatment alone. Furthermore, pretreatment with SP600125 markedly attenuated the upregulation of the pro-apoptotic protein Bax induced by S20 and H20.

#### 3.5.2 p38/JNK MAPK signaling pathways affect oxidative stress and mitochondrial membrane potential in H9c2 cells

**FIGURE 11:**
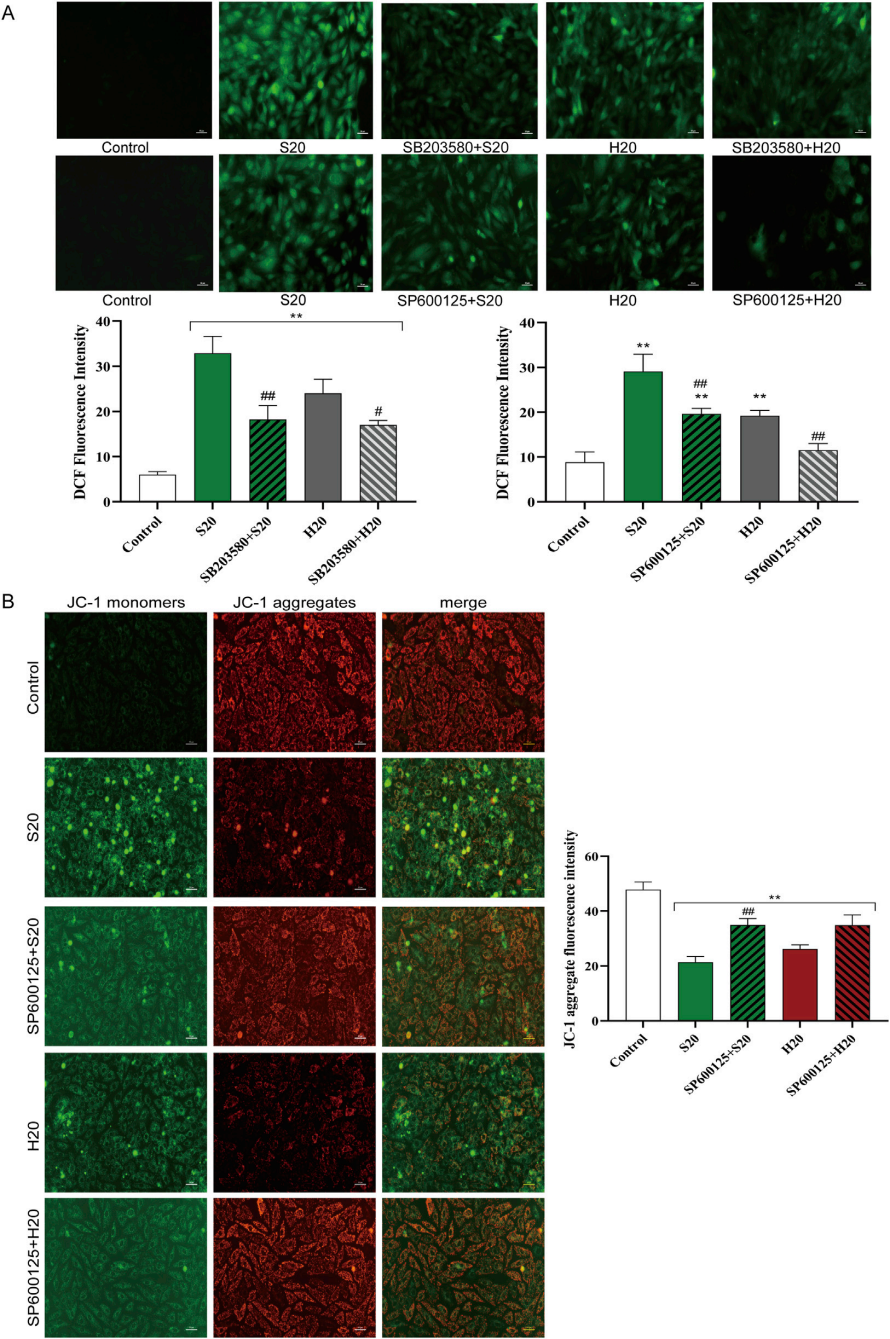
Effects of p38/JNK inhibition on SCW/HCW-induced ROS production and JNK blockade-mediated mitochondrial membrane potential changes in H9c2 Cells. **(A)** Effects of p38/JNK inhibition on SCW/HCW-induced ROS production in H9c2 Cells by DCFH-DA, fluorography (×100), quantification is expressed as the DCF fluorescence intensity. **(B)** Effect of JNK inhibition on SCW/HCW-induced mitochondrial membrane potential in H9c2 cells by JC-1 dye staining, fluorography (×100), quantification is expressed as the JC-1 aggregate fluorescence intensity (*x* ± *s, n* = 3. Compared with control, ^**^ *p* < 0.01; comparison between groups with and without p38/JNK inhibition, ^*##*^ *p* < 0.01, ^*#*^ *p* < 0.05).

ROS production in H9c2 cells treated with S20 or H20 in the presence or absence of SB203580 (a p38 inhibitor) or SP600125 (a JNK inhibitor) was examined. Concurrently, changes in mitochondrial membrane potential in cells treated with S20 or H20 in the presence or absence of SP600125 were assessed. Pretreatment with SB203580 or SP600125 for 1 h was found to prevent the increase in cellular ROS release caused by S20 and H20 treatment, indicating that the oxidative stress induced by S20 and H20 was associated with the activation of the p38 and JNK signaling pathways (Figure 11A). Pretreatment with SP600125 for 1 h was found to prevent the reduction in mitochondrial membrane potential induced by S20 and H20 treatment (Figure 11B), indicating that the decrease in mitochondrial membrane potential induced by S20 and H20 was mediated by the JNK signaling pathway.

## 4 Discussion

Mongolian medicine is an important part of TCM. CW, as a Mongolian medicine, has made a great contribution to the prevention and treatment of human diseases. Although CW possess excellent anti-inflammatory and analgesic properties, their toxicity is remarkably high (Singhuber et al., 2009). Modern research has shown that among the symptoms of poisoning caused by taking Aconitum botanical drugs, those of the cardiovascular system are particularly prominent (Tao et al., 2023a). It is usually manifested as overlapping occurrence of various life-threatening arrhythmias (Coulson et al., 2017). It is generally accepted that diterpenoid alkaloids are the major active metabolites of Aconitum botanical drugs. However, monoester-type diterpenoid alkaloids are the main hydrolysates of diester-type diterpenoid alkaloids, and they exhibit significantly reduced toxicity but not complete elimination. (Yang et al., 2018). According to the published literature, aconitine, mesaconitine, and hypaconitine are the alkaloid metabolites with the highest content in CW (Fu et al., 2024), and the anti-inflammatory effects and analgesic effects of them have been demonstrated by previous studies (Gao et al., 2022). However, the three metabolites are DA with fatal cardiotoxicity and neurotoxicity (Liu et al., 2017b). At present, the poisoning events caused by improper use of Aconitum botanical drug have happened frequently (Sitthiprawiat, et al., 2025). Hence, it is urgent to explore the scientific connotation of attenuation from the traditional medication experience of aconitum botanical drugs.

Traditionally, in addition to reducing the toxicity by reasonable processing methods, co-administering it with other medicinal excipients is a common way to reduce side effects. Different from rice vinegar and yellow rice wine, which are commonly used in TCM, Mongolian medicine excipients include HZ decoction, milk and highland barley wine. HZ plays a crucial role in Mongolian medicine, which is good at using it to reconcile various medicines. SCW is usually used in combination with HZ or soaked in HZ decoction to achieve the purpose of reducing toxicity and preserving efficacy (Li et al., 2019; Li H. et al., 2021). Strict control of alkaloid content is imperative for both CW and its processed products to ensure safe, effective clinical use. At present, the SCW and HCW obtained by the processing technology we have determined are qualified products in accordance with the pharmacopoeia. Different liquid chromatography methods are used in the Chinese Pharmacopoeia to determine the contents of DA and MA. The Processing specification of Inner Mongolia Mongolian medicine decoction pieces uses a single method to determine DA and MA; the required elution time of 85 min makes the method too lengthy and inconvenient for practical use. In addition, sustained-release dosage forms are also an effective experience of Mongolian medicine (Hu et al., 2024). From the perspective of dosage form, when administered as pills or powders, the release and absorption rate of the drug is lower than that of the decoction. As a result, the metabolites with both toxic and curative effects are released slowly in the body, maintaining the blood drug concentration at a low level, while reducing toxicity and ensuring continuous efficacy (Han, 2013). Clarifying the main metabolite contents of different dosage forms of SCW and HCW is the key to ensure the safety of clinical medication. Therefore, we established two efficient HPLC methods, each tailored for a specific dosage form, to simultaneously determine six ester-type alkaloids in the powder and water decoction of different processed products of CW. These methods aim to improve work efficiency and provide a reference for the quality control of Chinese patent medicines containing CW. We found that processing could reduce the content of total alkaloids. The addition of water for decoction would promote the hydrolysis of DA in the processed products of CW to MA. This finding conforms to the hydrolysis law of the alkaloids in CW and is also consistent with the requirement that Aconitum-related TCM often ought to be decocted first and for a long time in clinical application (Chan et al., 2021; Wang F. et al., 2025). After HZ decoction processing, there was still a large amount of DA in HCW, and the total alkaloid content in HD is relatively high compared to that in HP. This might be influenced by the changes in the physical and chemical properties of alkaloids and the impact exerted by the metabolite of HZ and the different dosage forms (Liu et al., 2013; Zhao et al., 2016; Zhi et al., 2020b).

The efficacy and toxicity of CW are the key factors to determine its clinical application. Therefore, it is necessary to comprehensively analyze the performance of its efficacy and toxicity. Studies have shown that CW has a significant effect in anti-inflammation and analgesia (Lai et al., 2012; An et al., 2014; Li H. et al., 2021). This is consistent with its clinical application. The mouse ear swelling and formalin pain experiments showed that each sample of CW had different degrees of anti-inflammatory and analgesic effects. And this effect may be related to inhibiting the release of pro-inflammatory factors (IL-1β, IL-6), pain factors (PGE_2_, 5-HT), and promoting the release of anti-inflammatory factor (IL-10). Furthermore, SP has excellent inhibitory effects on both acute and inflammatory pain, while HP has a better inhibitory effect on inflammatory pain. In addition, our results showed that the anti-inflammatory effect of the SD group was stronger than that of the SP group, while the analgesic effect of the SP group was better than that of the SD group. This difference may be due to the different types and contents of alkaloids in SP and SD, indicating that different types of alkaloids exert different biological activities, and further confirming that the form of administration will significantly affect the clinical efficacy of CW (Li Y. et al., 2021). Moreover, in terms of anti-inflammatory and analgesic effects, the effect of the HP group was better than that of the HD group, which indicates that it is reasonable for Mongolian medicine to use the HCW in the form of powder for making bolus and powder preparations.

CW is a highly toxic medicinal material, with the heart being its main toxic target organ. The toxic metabolites directly stimulate the heart after entering the bloodstream, leading to various premature beats or arrhythmias (Coulson et al., 2017; Li et al., 2024). In severe cases, it can even cause death. The liver and kidney are the main organs responsible for drug metabolism and excretion, and long-term use of toxic drugs can greatly affect their functions (Hagenbuch, 2010). Our results showed that SP can increase the heart rate of rats and easily induce arrhythmia in rats. The histopathological and serum biomarker results further confirmed the cardiotoxicity of SP, as well as its toxicity to the liver and kidney, which is consistent with the known toxicity of CW (Wu and Bao, 2024). In comparison, the toxic effect of SD was significantly reduced, which is in line with the traditional view that decoction can detoxify (Chan et al., 2021). The treatment with HCW had a certain detoxifying effect, but it did not completely eliminate the risk of cardiotoxicity, and it caused abnormalities in liver and kidney functions. Moreover, with the passage of the administration time, the cumulative effect of the drug may lead to changes in the heart rate. Clinically, the daily HCW dosage for adults is 0.25–0.50 g. According to body surface area conversion, the human equivalent doses corresponding to the rat doses of 0.12 g/kg and 0.24 g/kg are higher than clinical dosages, which suggests that the toxicity risk of conventional clinical medication is reduced. Judging from the levels of serum biomarkers, compared with HP, HD was more toxic to a certain extent. This may be related to the different dissolution and metabolism of the drug in the liver and kidney under different dosage forms (Qu et al., 2022). In addition, the detection results of the contents of MDA, SOD and CAT in the myocardial tissue showed that oxidative stress is involved in myocardial injury. Compared with the SP group, the HP group exhibited a certain antioxidant effect, indicating that the processing with HZ can reduce the level of oxidative damage to the heart, which may be related to the certain antioxidant capacity of HZ itself (Wang et al., 2024). On the contrary, compared with the HP group, the protective effect of the HD group on cardiac oxidative injury was weaker, and it may even exacerbate the oxidative stress injury of the heart to a certain extent. This may be related to the different chemical metabolites and properties of the powder and decoction dosage forms after HCW treatment, thus affecting the degree of oxidative stress in the heart. In different administration cycles, the heart rate and arrhythmia scores of rats in the administration group showed a dynamic trend with time, and these findings suggest that for patients with arthralgia who need long-term medication, it is recommended to conduct 24-h Holter monitoring regularly, especially in the initial stage of medication and during the dosage adjustment stage.

So far, researchers have been investigating the toxicity mechanisms of aconitum botanical drugs and the mechanisms by which processing reduces their toxicity—findings that provide theoretical support for the safe clinical application of aconitum botanical drugs (Dong et al., 2009; Zhou et al., 2013; Wang et al., 2023; Lv and Jiao, 2024). Among these toxicity mechanisms, oxidative damage plays a crucial role in the development of cardiac toxicity. In normal organisms, a small amount of ROS is produced to conduct signals in cells, and ROS is easily scavenged by the antioxidant defense system. Oxidation and antioxidant reactions restrict each other and maintain cell homeostasis. In the heart, excessive production of ROS exceeds the ability of the antioxidant defense system to withstand. This imbalance is precisely what occurs when aconitum botanical drugs induce cardiac toxicity. Oxidation and anti-oxidation are out of balance, and oxidative stress occurs immediately, leading to myocardial cell damage or even death, resulting in a series of cardiovascular diseases (Wang et al., 2017; van der Pol et al., 2019). SOD is an important antioxidant enzyme, and GSH is an antioxidant-related substance. Their contents, together with the content of MDA (the end product of lipid peroxidation, which can indirectly reflect the degree of oxidative damage in the body), can reflect the degree of antioxidant capacity and cell damage to a certain extent. LDH content represents the process of cell damage (Zhao et al., 2018; Wang et al., 2022). Alkaloids in Aconitum can reduce the content of SOD, resulting in weakened ability to remove oxidative factors (Ma et al., 2018). In our results, SP and HP also stimulated ROS levels and led to cell damage and cell death, consistent with the previous description (He, 2023). Therefore, reducing this imbalance of oxidative stress is one of the possible ways to reduce the cardiotoxicity. In this study, we demonstrated that SP can damage H9c2 cardiomyocytes by inducing oxidative stress, which in turn destroys mitochondrial membrane potential and triggers apoptosis, eventually leading to H9c2 cardiomyocyte damage. In contrast, the cardiotoxicity of HP is relatively weak, and low-dose HP may even have a protective effect on cardiomyocytes. From the changes of oxidative stress-related indicators, the degree of oxidative damage caused by HP was significantly lighter than that of SP, which may be one of the factors for the weaker myocardial toxicity of HP. Specifically, after exposure to SP and HP, there were significant differences in the changes of ROS level, MDA content and antioxidant enzyme activities such as SOD and GSH in cardiomyocytes, which further confirmed the differences in cardiotoxicity between SP and HP. The cell experiment excluded the interference of physiological environment, and the results further verified the conclusion of the above animal experiment. He Miao (He, 2023) showed that aconitine combined with glycyrrhetinic acid can inhibit the oxidative damage caused by aconitine and protect the mitochondrial structure, and our previous studies have also shown that gallic acid and ellagic acid can reduce the toxic effects of mesaconitine and benzoylmesaconitine (Han et al., 2022). These acidic metabolites can affect the absorption, distribution, and metabolism of alkaloids *in vivo*, and reduce the effect of toxic metabolites on target organs. And these metabolites have certain antioxidant activity, which may be one of the important mechanisms to reduce the toxicity of aconitine drugs (Pastorino et al., 2018; Bobasa et al., 2021).

Because of these changes, oxidative damage of cardiomyocytes seems to be the main mechanism of apoptosis of H9c2 cardiomyocytes by SP and HP treatment. Proteins in MAPK signaling pathways such as p38 and JNK kinases are activated in response to ROS generation (Sui et al., 2014; Zeke et al., 2016; Zhao et al., 2016; Sahoo et al., 2022). The p38 and JNK signaling pathways can lead to cell necrosis and apoptosis in stress responses such as inflammation and other stress responses (Renaudin, 2021). Furthermore, ROS accumulation plays a key role in mitochondrial-associated apoptotic cell death in various physiological conditions (Moloney and Cotter, 2018). Apoptosis is known to be regulated by disruption of redox homeostasis in cells (Shirmanova et al., 2022). Moreover, JNK can directly regulate the key proteins of mitochondrial apoptosis, and can quickly induce the collapse of mitochondrial membrane potential under stress, which is more closely related to mitochondrial membrane potential (Win et al., 2018). Here, we explored heart toxicity mechanisms of SCW and HCW involving ROS-dependent mitochondrial dysfunction, which led to growth inhibition and apoptosis of H9c2 cells. In this study, SCW broke the balance of oxidation and antioxidant system *in vivo*, induced oxidative stress in rat heart, and produced cardiotoxicity. The expression of p38, JNK signaling pathway and apoptosis-related proteins in cardiomyocytes was further detected. The results showed that compared with the control group, the phosphorylation levels of p38 and JNK and the expression of Bax in the SCW group were significantly increased, and the expression of Bcl-2 was significantly decreased. The p38 MAPK and JNK protein kinases are activated, and the MAPK signaling pathway cascades. The activated p38 MAPK and JNK participate in the regulation of apoptosis by phosphorylating downstream substrates. SCW and HCW induced apoptosis *via* the mitochondrial signaling pathway by up-or down-regulating Bcl-2 family proteins. In addition, HCW increased the phosphorylation levels of p38 and JNK at each dose, indicating that the metabolites in HZ could be introduced in a large amount when processing SCW, and could have a synergistic effect with the metabolites of SCW itself. Previous studies on the characteristic spectra and mass spectrometry analysis of HCW can provide relevant evidence (Liu et al., 2017a; Zhi et al., 2020a). Compared with S10 and S20 groups, the expression level of Bax in H10 and H20 groups was significantly decreased. Although both S20 and H20 induced cytotoxicity through activating the p38/JNK MAPK pathway, the toxicological profile of HP was notably different from that of SP. The inability of the JNK inhibitor to rescue Bcl-2 expression in SP-treated cells suggests the involvement of additional, more robust cytotoxic mechanisms. In contrast, the increasing trend of Bcl-2 expression in HP-treated cells upon JNK inhibition indicates that its toxicity is more reliant on the JNK pathway and is consequently more reversible. This higher reversibility and a more confined mechanism of action underpin the reduced cardiotoxicity of HP compared to SP. The results showed that HCW could also affect cell damage by regulating the activation of p38/JNK MAPK, and may reduce myocardial toxicity by the JNK-mediated mitochondrial apoptosis pathway.

### 4.1 Limitations and future directions

However, several limitations of this study should be acknowledged. Firstly, in terms of experimental design, we only used the suspension solvent of the powder sample as the control. Including an additional water control group would have further enhanced the rigor of the experiment, and this oversight represents a methodological limitation. Secondly, considering that TCM is a complex chemical system, we speculate that in addition to the alkaloids already quantified in this paper, which are specified in the pharmacopoeia, other unexamined metabolites may indirectly participate in the regulation of CW efficacy and toxicity—either by affecting the absorption, distribution or metabolism of core alkaloids *in vivo* or by directly acting on other targets. This reflects the typical “multi-metabolite, multi-target” mode of action of TCM; however, in-depth investigation into this aspect is still lacking in the current research, and the effect of its metabolites after entering the bloodstream also requires further exploration.

In the future, the regulatory effect of non-core metabolites on the efficacy and toxicity of core metabolites can be systematically evaluated through “metabolite knockout/addition” experiments or integrated pharmacology approaches. Additionally, association analysis of metabolites with their biological effects and mechanisms using multi-omics techniques. These approaches will not only more comprehensively reveal the overall mechanism of CW but also provide a more holistic framework for studying the efficacy and toxicity of complex TCM systems.

## 5 Conclusion

Processing methods and dosage forms affect the chemical composition content, efficacy, and toxic effects of CW. The findings of this study suggest that: 1) SCW exhibits anti-inflammatory and analgesic effects but carries a risk of cardiac toxicity, which is consistent with previous studies; HZ decoction treatment or water decoction treatment can change the content of alkaloids, and these processed products show lower toxicity than SCW while retaining some pharmacological activities; 2) HP: The clinical routine dose is effective, sometimes the effect can be equivalent to SP, and the analgesic effect is stable compared with HD; when the dose is excessive, its toxicity is lower than that of SP at the same dose, and exhibits lower toxicity than HD in some biochemical indicators; because of the small dosage of HP in clinical practice, the dose of HP compared with SP retains the therapeutic effect and reduces the toxicity, which is consistent with the practice of Mongolian medicine using toxic plant drugs mainly in pills or powder dosage forms and using small doses of drugs; 3) The p38/JNK MAPK pathway and mitochondrial apoptotic pathway are likely key molecular mechanisms mediating these toxic effects.

## Data Availability

The original contributions presented in the study are included in the article/supplementary material, further inquiries can be directed to the corresponding author. Raw data was uploaded to FigShare and is available at the following link: https://figshare.com/s/3fc91ab9855e6d9e125e (Doi: 10.6084/m9.figshare.30186562).

## Glossary

5- HT: 5-Hydroxytryptamine
ALT: alanine transaminase
AST: aspartate transaminase
AT: atrial tachycardia
BUN: blood urea nitrogen
CAT: catalase
CK: creatine kinase
Cr: creatinine
CTnⅠ: cardiac troponin I
CW: Aconiti Kusnezoffii Radix
ECG: electrocardiogram
ELISA: enzyme-linked immunosorbent assay
GSH: glutathione
HCW: Aconitum kusnezoffii processed with Chebulae Fructus
HD: decoction of HCW
HE: Hematoxylin-Eosin staining
HP: powder of HCW
HPLC: high performance liquid chromatography
HR: heart rate
HZ: Chebulae Fructus
IL-1β: interleukin-1β
IL-6: interleukin-6
IL-10: interleukin-10
JNK: c-Jun N-terminal kinase
LDH: lactate dehydrogenase
MAPK: mitogenactivated protein kinase
MDA: malondialdehyde
MTT: 3-(4,5-dimethylthiazol-2-yl)-2,5 diphenyltetrazolium bromide
NSR: normal sinus rhythm
NT-proBNP: n-terminal pro-brain natriuretic peptide
p38: p38 mitogen-activated protein kinase
**PGE**_**2**_: prostaglandin E2
PVCs: premature ventricular contractions
RA: rheumatoid arthritis
ROS: reactive oxygen species
RSD: relative standard deviation
SCW: raw Aconiti Kusnezoffii Radix
SD: decoction of SCW
SDS-PAGE: sodium dodecyl sulfate polyacrylamide gel electrophoresis
SOD: superoxide dismutase
SP: powder of SCW
TCM: Traditional Chinese Medicine
TdP: torsade de pointes
VF: ventricular fibrillation
VT: ventricular tachycardia
Wb: Western blotting
WGR: weight growth rate
