# Supplementary material for: Mongolian medicine theory-based multidimensional evaluation of toxicity mitigation in Hezi-processed Caowu jointly mediated by powder dosage form and small dose
Source: Front Pharmacol. 2025 Oct 1;16:1679105. doi: 10.3389/fphar.2025.1679105 (PMC12521102; doi:10.3389/fphar.2025.1679105)
Supplement: Supplementary file 1 [file Supplementaryfile1.docx]

Supplementary Material

Mongolian Medicine Theory-Based Multidimensional Evaluation of Toxicity Mitigation in Hezi-Processed Caowu Jointly Mediated by Powder Dosage Form and Small Dose

Jing Wang, Liyuan Bao, Ying Zhao, Liangliang Song, Wenting Zu, Jiasheng Wang, Hongshuang Chi, Yichen Li and Hong Du^*^

^1^School of Chinese Materia Medica, Beijing University of Chinese Medicine, Beijing, China

*** Correspondence:** Hong Du: duhong@vip.163.com

# Appendix Ⅰ. Molecular docking study of triterpenoids, mesaconitine and MAPK pathway receptors

The preparation of ligands and the acquisition and processing of MAPK signaling pathway-related protein receptors refer to the previous methods of the research group [Song et al., 2024]. The eight MAPK signaling pathway-related core proteins receptors that we selected for molecular docking were downloaded from the Protein Data Bank (PDB) database (http://www.ncbi.nlm.nih.gow/protein/). They are respectively the structure of ERK2 (PDB ID code: 1PME; Resolution: 2.0Å), the structure of p38 MAPK (PDB ID code: 1YQJ; Resolution: 2.0Å), the structure of JNK2 (PDB ID code: 3E7O; Resolution: 2.14Å), the structure of AKT1 (PDB ID code: 4EJN; Resolution: 2.19Å), the structure of FGFR3 (PDB ID code: 4K33; Resolution: 2.34Å), the structure of ERK1 (PDB ID code: 6GES; Resolution: 2.07Å), the structure of JNK1 (PDB ID code: 4G1W; Resolution: 2.45Å), and the structure of RAP1A (PDB ID code: 1GUA; Resolution: 2.0Å).

Semi-flexible molecular docking was performed using AutoDock Vina, with protein receptors kept rigid while allowing rotatable bonds in small-molecule ligands. We needed to pretreat the protein receptors, including dehydration, hydrogenation, etc. Co-crystallized ligands were extracted, and cubic docking grids were centered on their coordinates with edge lengths optimized for each receptor: 1YQJ (X = 22.587, Y = 12.626, Z = 31.666, side length =20.006) [Fox et al., 1998], 1PME (X = -12.976, Y = 13.195, Z = 40.562, side length = 19.718) [Tamayo et al., 2005], 3E7O (X = -30.942, Y = -32.680, Z = 23.934, side length = 18.109) [Shaw et al., 2008], 4EJN (X = 35.389, Y = 43.721, Z = 18.443, side length = 24.046) [Ashwell et al., 2012], 4K33 (X = 27.607, Y = 3.267, Z = 35.815, side length = 15.939) [Huang et al., 2013], 6GES (X = 61.256, Y = 21.141, Z = 18.460, side length = 18.199) [Rao et al., 2019], 4G1W (X = -1.415, Y = 0.573, Z = -10.807, side length = 19.924), 1GUA (X = -2.998, Y = 26.804, Z = 26.361, side length = 21.287) [Nassar et al., 1996].

The co-crystallized ligands were redocked, and their poses were superimposed with the original structures to calculate RMSD values, as follows: RMSD (1PME) = 0.8437Å, RMSD (1YQJ) = 0.2565Å, RMSD (3E7O) = 1.1495Å, RMSD (4EJN) = 0.4663Å, RMSD (4K33) = 1.8091Å, RMSD (4G1W) = 1.0379Å, RMSD (1GUA) = 0.4603Å, RMSD (6GES) = 0.9486Å. All RMSD values were < 2Å, indicating that the redocked structures closely matched the original ligands in both conformation and spatial position, which validated the reliability of the docking protocol. Subsequently, seven triterpenoid components and mesaconitine were docked into the aforementioned receptors, and the results were exported.

It can be seen from Table S1 that mesaconitine has a certain affinity for each protein receptor of MAPK signaling pathway, among which the affinity with AKT1, EKR2, EKR1 and p38 MAPK is the strongest, indicating that mesaconitine has a high potential to bind to these four proteins. And Triterpenoids have high affinity with these eight proteins, among which ERK1, ERK2 and p38 MAPK have the strongest affinity, indicating that triterpenoids are most likely to bind to these three proteins.

The schematic diagram of the binding of mesaconitine to these eight protein receptors is shown in Figure S1. The benzene ring of mesaconitine interacts with multiple proteins and can interact with multiple protein residues, mainly π-alkyl and hydrogen bonds, indicating that the benzene ring may be the key structure for mesaconitine to play a role through the MAPK signaling pathway.

**Table S1.** Docking scores of mesaconitine and the selected triterpenoids with protein receptors of MAPK signaling pathway (Kcal/mol).

|  | 1GUA | 1YQJ | 3E7O | 4EJN | 4G1W | 4K33 | 6GES | 1PME |
| --- | --- | --- | --- | --- | --- | --- | --- | --- |
| Mesaconitine | -6.4 | -6.9 | -4.9 | -7.4 | -6.4 | -3.8 | -6.9 | -7 |
| Corosolic acid | -7.4 | -9.9 | -7.5 | -10.4 | -7.3 | -7.4 | -8.7 | -8.2 |
| Maslinic acid | -6.1 | -8.6 | -6.5 | -7.1 | -7 | -6.5 | -8.5 | -7.9 |
| Arjunolic acid | -6.3 | -9.7 | -5.7 | -8.5 | -7.2 | -7.8 | -7.6 | -7.7 |
| Chebupentol | -6.3 | -5.8 | -4.5 | -6.8 | -6.1 | -3.8 | -6.5 | -7.4 |
| Chebuloside-Ⅱ | -8.1 | -8.5 | -6.5 | -10 | -8.3 | -5.9 | -8.3 | -8.4 |
| Terminolic acid | -6.7 | -9.3 | -6 | -8.2 | -6.6 | -6.1 | -8 | -7.5 |
| Arjungenin | -6.3 | -9.4 | -5.9 | -8.4 | -7.3 | -6.5 | -7.6 | -7.7 |

Note: The smaller the value, the higher the affinity to the protein receptor.

RAP1A (PDB ID code: 1GUA); p38 MAPK (PDB ID code: 1YQJ); JNK2 (PDB ID code: 3E7O); AKT1 (PDB ID code: 4EJN); JNK1 (PDB ID code: 4G1W); FGFR3 (PDB ID code: 4K33); ERK1 (PDB ID code: 6GES); ERK2 (PDB ID code: 1PME)
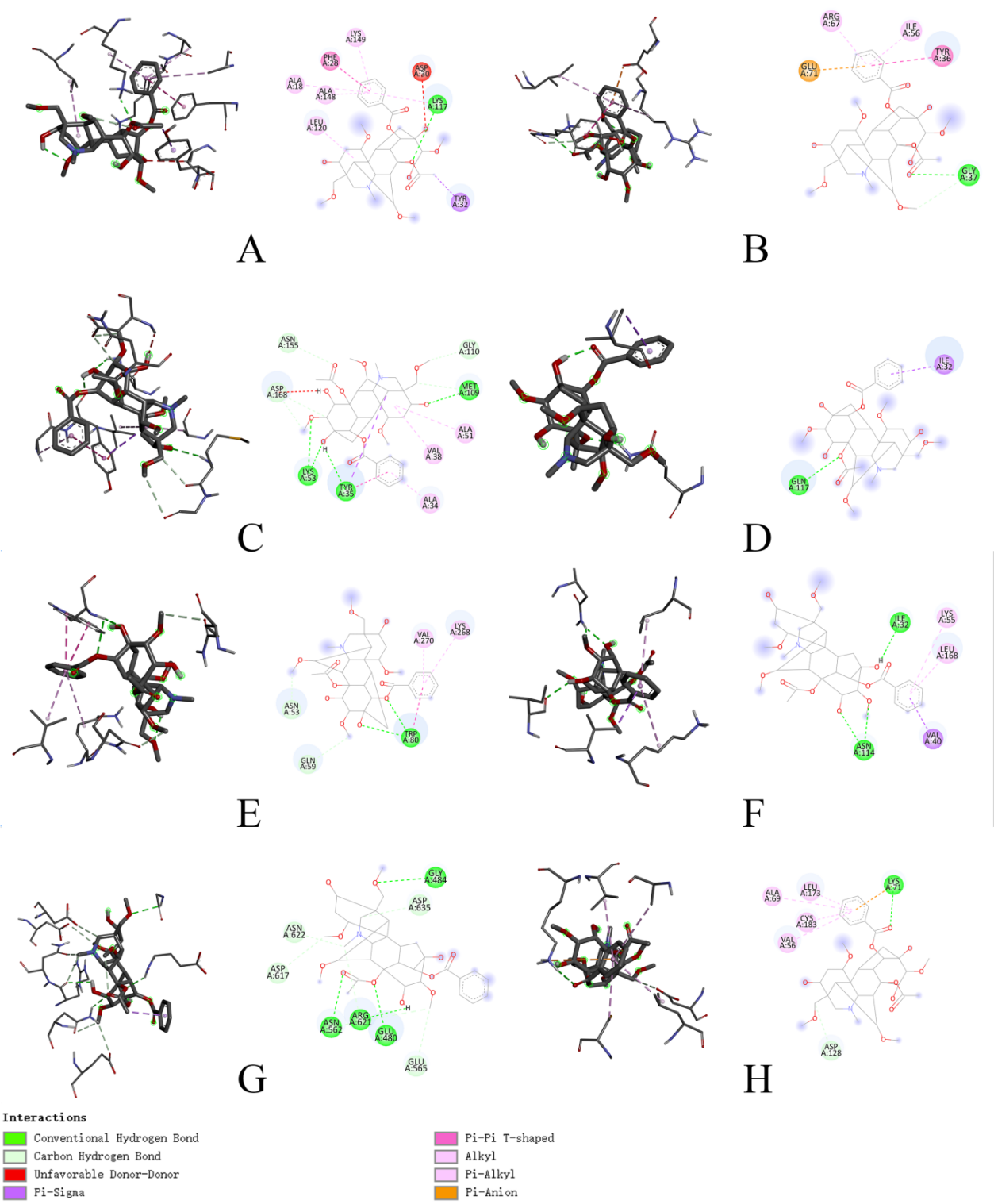


**FIGURE S1.** Schematic diagram of docking binding of mesaconine with protein receptors in MAPK signaling pathway. A：RAP1A；B：ERK2；C：p38 MAPK；D：JNK2；E：AKT1；F：JNK1；G：FGFR3；F：ERK1.

**Reference**

Ashwell MA, Lapierre JM, Brassard C, Bresciano K, Bull C, Kennon SC, et al. (2012). Discovery and optimization of a series of 3-(3-phenyl-3H-imidazo[4,5-b]pyridin-2-yl)pyridin-2-amines: orally bioavailable, selective, and potent ATP-independent Akt inhibitors. *J Med Chem*;55:5291-310.

Fox T, Coll JT, Xie X, Ford PJ, Germannet UA, Porter MD, et al. (1998). A single amino acid substitution makes ERK2 susceptible to pyridinyl imidazole inhibitors of p38 MAP kinase. *Protein Sci*. 7:2249-55.

Huang Z, Chen H, Blais S, Neubert TA, Li X, Mohammadi M. (2013). Structural mimicry of a-loop tyrosine phosphorylation by a pathogenic FGF receptor 3 mutation. *Structure,* 21:1889-96.

Nassar N, Horn G, Herrmann C, Block C, Janknecht R, Wittinghofer A. (1996). Ras/Rap effector specificity determined by charge reversal. *Nat Struct Biol*;3:723-9

Rao S, Gurbani D, Du G, Everley RA, Browne CM, Chaikuad A, et al. (2019). Leveraging Compound Promiscuity to Identify Targetable Cysteines within the Kinome. *Cell Chem Biol*;26:818-829.e9.

Shaw D, Wang SM, Villaseñor AG, Tsing S, Walter D, Browner MF et al. (2008). The crystal structure of JNK2 reveals conformational flexibility in the MAP kinase insert and indicates its involvement in the regulation of catalytic activity. *J Mol Biol*;383:885-93.

Song L, Mi S, Zhao Y, Liu Z, Wang J, Wang H, et al. Integrated virtual screening and in vitro studies for exploring the mechanism of triterpenoids in Chebulae Fructus alleviating mesaconitine-induced cardiotoxicity via TRPV1 channel. *Front Pharmacol* 2024;15:1367682.

Tamayo N, Liao L, Goldberg M, Powerset D, Tudoral YY, Yu V, et al. (2005). Design and synthesis of potent pyridazine inhibitors of p38 MAP kinase. *Bioorg Med Chem Lett*;15:2409-13.

# Appendix Ⅱ. Examples of arrhythmia scoring criteria (Rats).


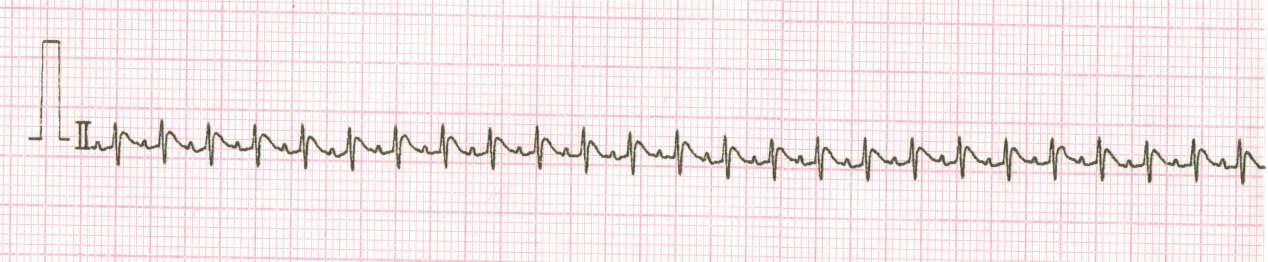


**FIGURE S2.** No arrhythmia, 0 points.


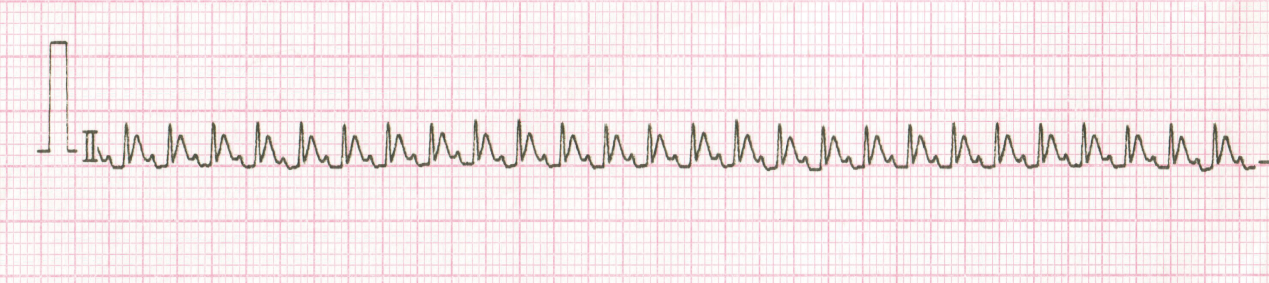


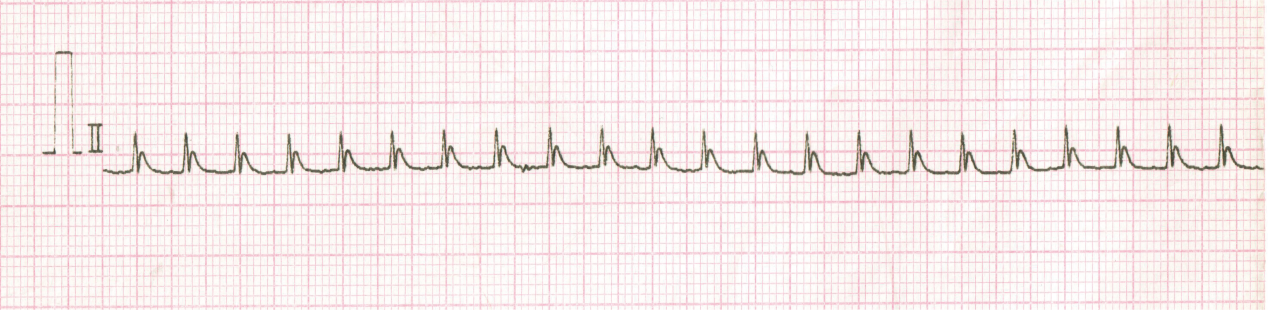


**FIGURE S3.** Atrial arrhythmia, 1 point.


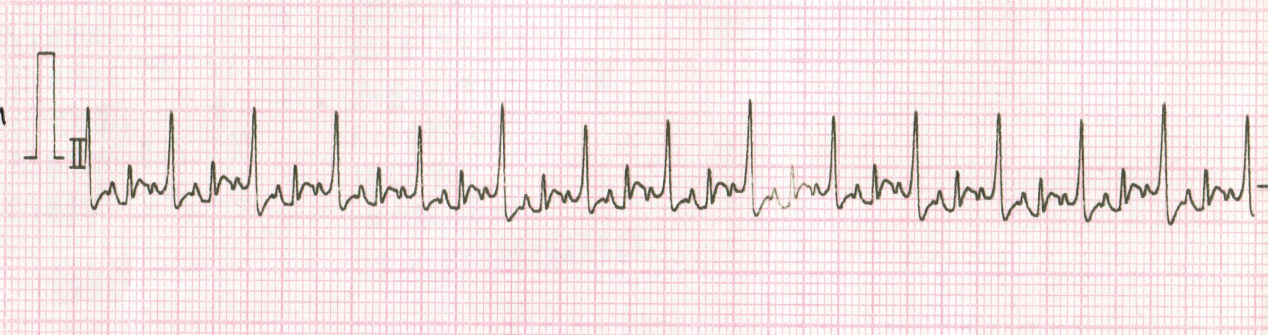


**FIGURE S4.** Occasional ventricular premature beats, 2 points.


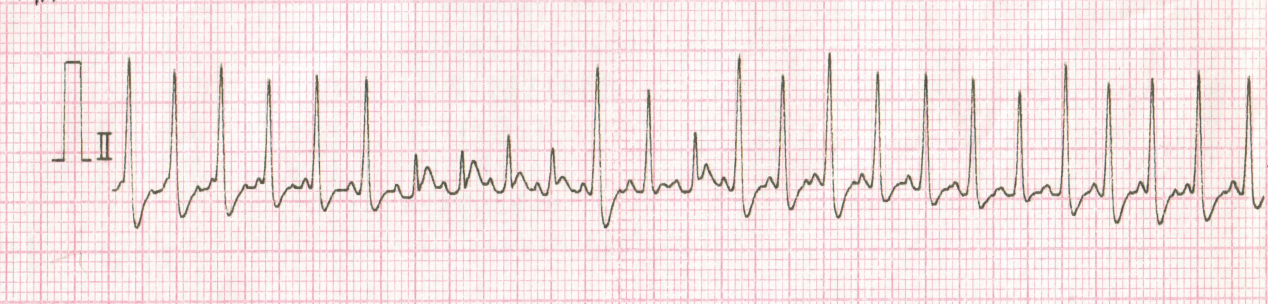


**FIGURE S5.** Frequent ventricular premature beats, 3 points.


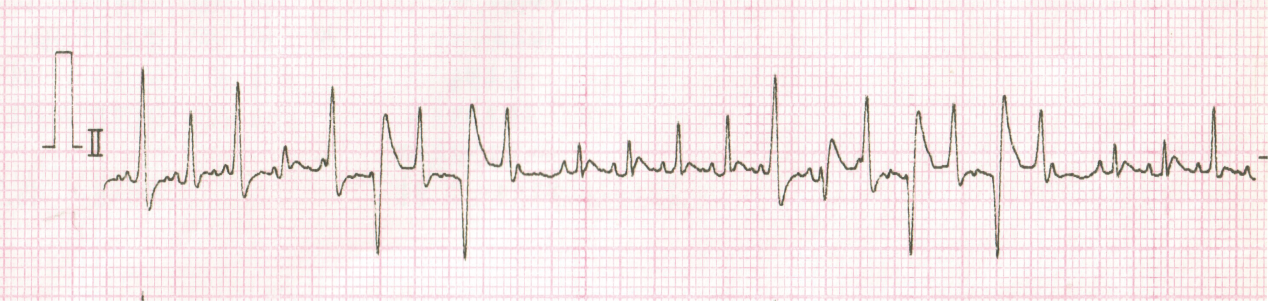


**FIGURE S6.** Incidental ventricular tachycardia, 4 points.


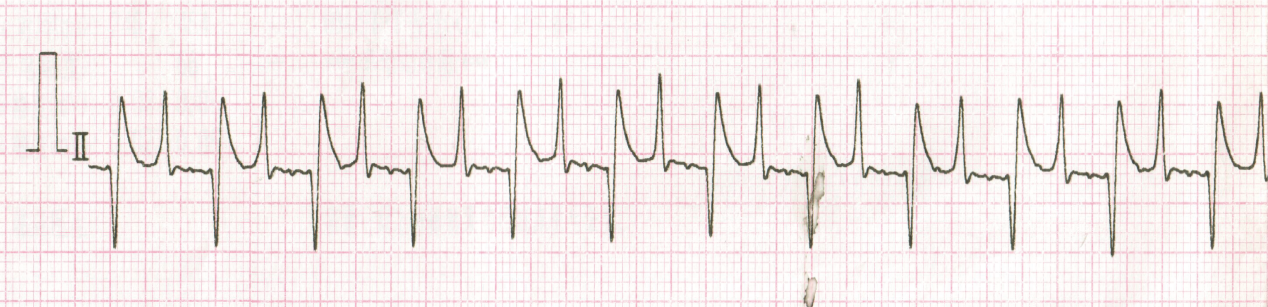


**FIGURE S7.** Frequent ventricular tachycardia or occasional ventricular fibrillation, 5 points.


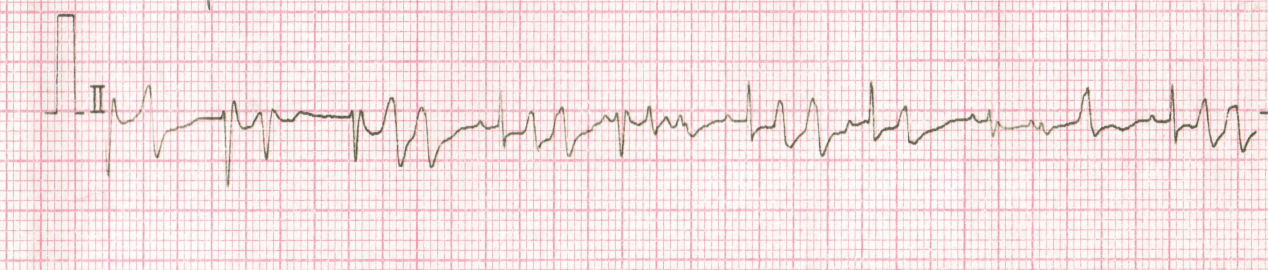


**FIGURE S8.** Persistent ventricular fibrillation or death, 6 points

# Supplementary Tables

**Table S2.** Results of linear relationship investigation of the HPLC method.

| Component | linear equation (range of linearity) |
| --- | --- |
| benzoylmesaconine | A: Y=10997X+390.03 (r=0.9999; X: 3.12~624.00μg/mL)  B: Y=10742X+36626 (r=0.9999; X: 3.58~716.00μg/mL) |
| benzoylaconine | A: Y=12408X-5006.3 (r=0.9999; X: 3.05~305.00μg/mL)  B: Y=11608X+10139 (r=0.9999; X: 2.18~436.00μg/mL) |
| benzoylhypaconine | A: Y=12963X-13608 (r=0.9999; X: 3.98~398.00μg/mL)  B: Y=11233X+8377 (r=0.9999; X: 2.15~430.00μg/mL) |
| mesaconitine | A: Y=12178X-688.03 (r=1.0000; X: 3.09~618.00μg/mL)  B: Y=11719X+16630 (r=0.9998; X: 1.82~364.00μg/mL) |
| hypaconitine | A: Y=12429X-7710.7 (r=0.9999; X: 4.31~431.00μg/mL)  B: Y=11998X+5520.6 (r=0.9999; X: 1.57~314.00μg/mL) |
| aconitine | A: Y=11930X-11012 (r=0.9999; X: 3.08~308.00μg/mL)  B: Y=12418X+790.91 (r=0.9999; X: 2.28~456.00μg/mL) |

Note: A, the linearity of HPLC method for SCW/HCW powder extract; B, the linearity of HPLC method for SCW/HCW water decoction extract. SCW: raw Aconiti Kusnezoffii Radix; HCW, Aconitum kusnezoffii processed with Chebulae Fructus.

**Table S3.** The precision, repeatability, and stability of the HPLC method.

| Component | RSD for precision | | RSD for repeatability | | RSD for stability | | Average recovery rate | | RSD for Recovery rate | |
| --- | --- | --- | --- | --- | --- | --- | --- | --- | --- | --- |
|  | A | B | A | B | A | B | A | B | A | B |
| benzoylmesaconine | 1.07% | 0.74% | 2.58% | 2.05% | 1.18% | 0.99% | 104.53% | 101.83% | 1.29% | 1.12% |
| benzoylaconine | 1.25% | 1.19% | 0.93% | 3.52% | 2.45% | 0.99% | 98.17% | 105.79% | 1.34% | 2.83% |
| benzoylhypaconine | 1.22% | 0.58% | 0.94% | 2.48% | 2.21% | 1.21% | 99.86% | 102.15% | 2.80% | 0.92% |
| mesaconitine | 0.95% | 1.70% | 1.74% | 3.69% | 1.03% | 4.39% | 101.45% | 104.00% | 2.06% | 2.21% |
| hypaconitine | 0.87% | 1.41% | 1.15% | 2.49% | 0.61% | 2.66% | 102.74% | 96.65% | 2.84% | 2.82% |
| aconitine | 0.84% | 1.96% | 1.39% | 1.15% | 1.50% | 2.05% | 103.41% | 103.48% | 1.31% | 1.71% |

Note: A, the HPLC method for SCW/HCW powder extract; B, the HPLC method for SCW/ZCW water decoction extract. SCW: raw Aconiti Kusnezoffii Radix; HCW, Aconitum kusnezoffii processed with Chebulae Fructus

# Appendix Ⅲ. Original blots of Figure 10


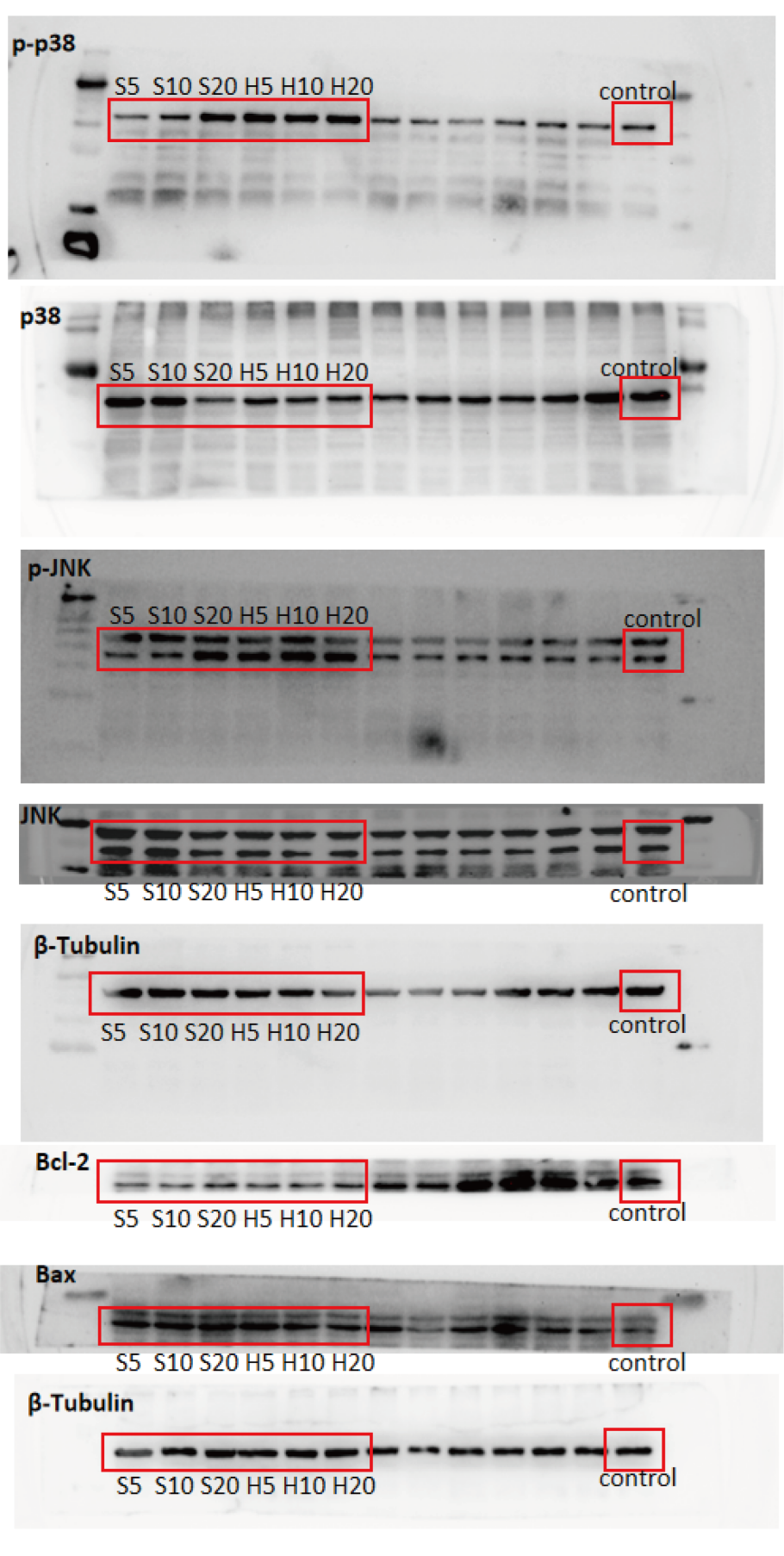


**Figure S9** Wb analysis of p-p38/p38, p-JNK/JNK, Bcl-2 and Bax expression levels. The H9c2 cells were treated with SCW/HCW powder (SP, HP) at 5, 10, or 20 mg/mL.


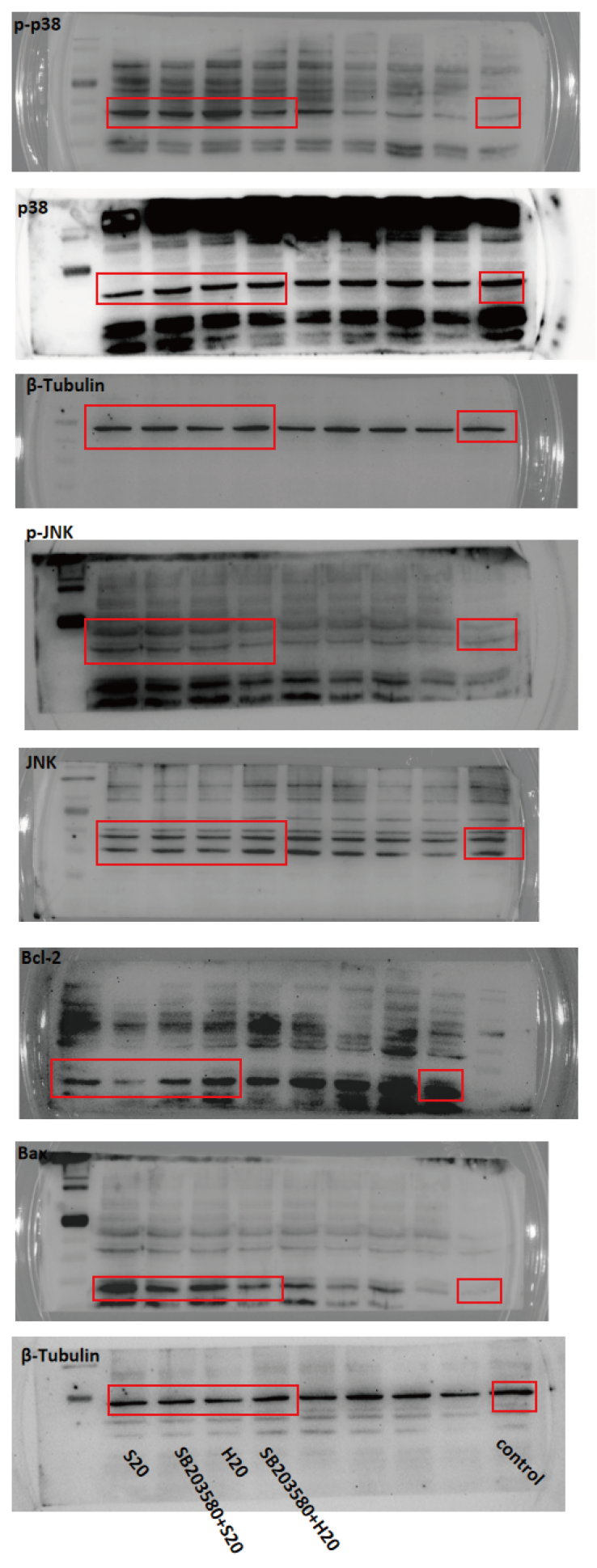


**Figure S10** Wb analysis and quantitation of p-p38/p38, p-JNK/JNK, Bcl-2, and Bax expression in H9c2 cells with or without p38/JNK inhibition. Cells were pretreated with p38 (SB203580) and JNK (SP600125) inhibitor for 1 h and then treated with 100 μL 20 mg/mL SCW or HCW powder for 24 h..
